# RBM42 couples alternative splicing to cytoskeletal organization to direct cortical development

**DOI:** 10.1016/j.isci.2026.118059

**Published:** 2026-11-02

**Authors:** Bingxin Yang, Qingxin Xu, Weiliang Lu, Xiang Qiu, Yanlu Chen, Runqian Shi, Yiyao Chen, Yuwu Jiang, Yanlin Wang, Jian Wang, Hong Xu, He-Feng Huang, Jinglan Zhang

**Affiliations:** 1 The International Peace Maternity and Child Health Hospital, School of Medicine, Shanghai Jiao Tong University, Shanghai 200030, China; 2 Shanghai Key Laboratory of Embryo Original Diseases, Shanghai 200030, China; 3 Institute of Medical Genetics and Development, Key Laboratory of Reproductive Genetics (Ministry of Education) and Women’s Hospital, Zhejiang University School of Medicine, Hangzhou, Zhejiang 310058, China; 4 Institute of Reproduction and Development, Shanghai Key Laboratory of Reproduction and Development, Obstetrics and Gynecology Hospital, Fudan University, Shanghai, China; 5 Children’s Medical Center, Peking University First Hospital, No. 5 Le Yuan Road, Daxing District, Beijing 102627, China; 6 Research Units of Embryo Original Diseases, Chinese Academy of Medical Sciences (No. 2019RU056), Shanghai, China; 7 School of Medicine, Shanghai Jiao Tong University, Shanghai, China

**Keywords:** alternative splicing, RBM42, cytoskeleton, cortical development

## Abstract

Alternative splicing coordinates gene expression programs that shape the developing cerebral cortex. Here, we used nervous system-specific *Rbm42* conditional knockout mice, RBM42-depleted human SH-SY5Y cells, RNA sequencing, RIP-seq, proteomics, and splice-switching rescue to define how the spliceosomal RNA-binding protein RBM42 supports cortical development. Rbm42 loss caused perinatal lethality, cortical thinning, defective lamination, reduced progenitor proliferation, increased apoptosis, and disrupted neuronal cytoskeletal organization. RBM42 bound and regulated cytoskeleton-associated transcripts, including *NIN*/*Nin* and *NUMA1*/*Numa1*, and its loss altered their splicing in human cells and mouse cortex. Restoring RBM42 corrected these splicing defects and improved proliferation and neurite growth. Selective correction of *NIN* exon 18 mis-splicing partially restored βIII-tubulin organization and proliferation but did not rescue apoptosis. These findings connect spliceosome-dependent isoform control with cytoskeletal remodeling during cortical development and provide a mechanistic framework for RBM42-associated neurodevelopmental disease.

## Highlights

• *Rbm42* loss causes cortical malformations and perinatal lethality in mice
• RBM42 regulates cytoskeletal genes with AS changes across species
• RBM42 loss alters *NIN* and *NUMA1* splicing and disrupts microtubule organization
• Correcting *NIN* exon 18 splicing partly rescues cytoskeletal and proliferative defects

## Graphical abstract

**Figure:**
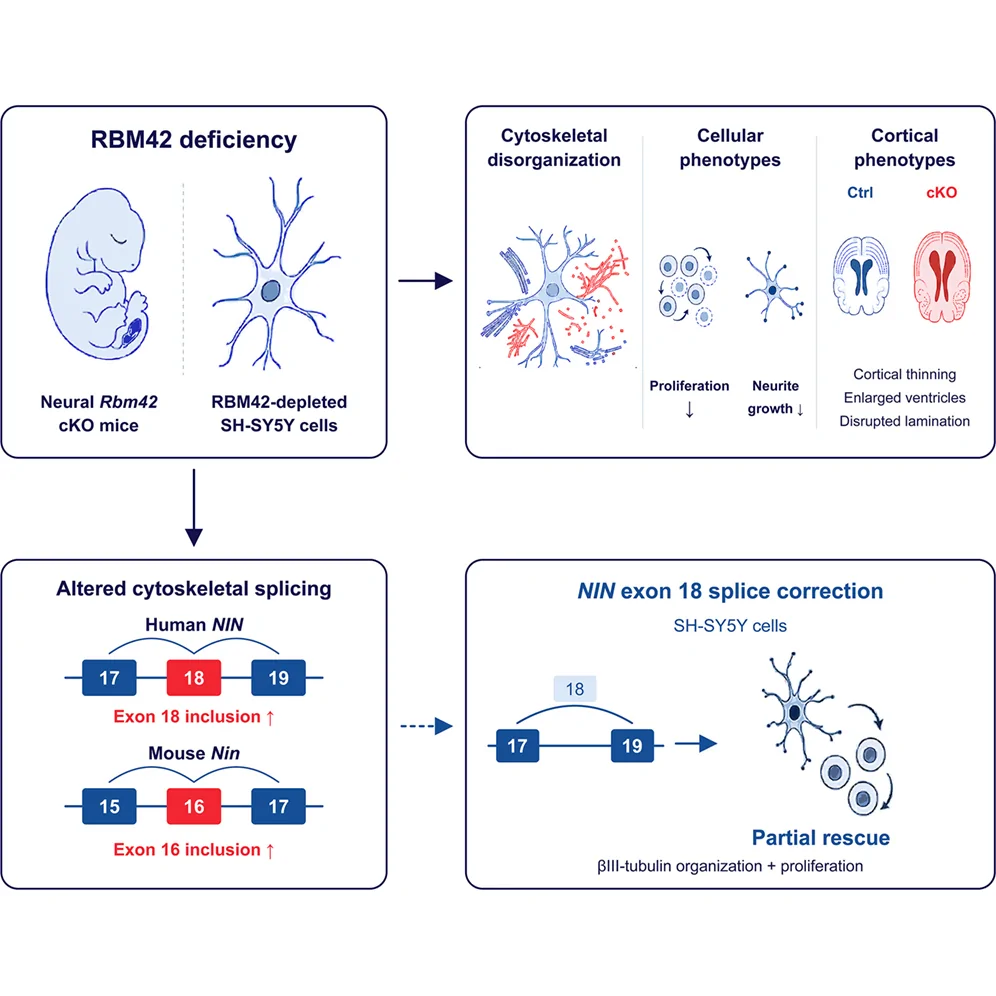

## Teaser

Neuroscience; Developmental neuroscience; Cell biology; Cell

## Introduction

Neurodevelopmental disorders (NDDs) arise from disruptions in the tightly regulated molecular programs that guide brain development.^1^^,^^2^ The etiologies of NDDs are highly heterogeneous and reflect contributions from genetic variation, epigenetic dysregulation, and environmental risk factors.^3^ Rare genomic variation can influence cognition, psychopathology, and brain structure,^4^ whereas prenatal environmental exposures such as maternal anemia have also been associated with NDD risk.^5^ Increasing evidence demonstrates aberrant alternative splicing (AS) as a key contributor to NDDs.^6^^,^^7^^,^^8^^,^^9^^,^^10^^,^^11^ Developmentally dynamic AS is particularly prevalent in the brain, supporting its cellular and functional diversity.^12^ Such splicing dynamics enable transcriptome remodeling essential for neurogenesis, neuronal migration, synaptogenesis, and circuit formation.^13^^,^^14^ Disruption of this regulatory layer impairs neuronal specification and connectivity, contributing to NDD pathogenesis. For instance, *HNRNPC* haploinsufficiency alters splicing of numerous intellectual disability-associated (ID-associated) genes, disrupting neuronal arborization and migration.^15^ Similarly, *HNRNPU* loss-of-function changes AS of genes critical for cell survival, motility, and synapse formation, leading to rapid death of both postmitotic neurons and neural progenitors.^16^ These studies highlight the acute sensitivity of the developing brain to perturbations in splicing regulators.

Central to the regulation of AS are RNA-binding proteins (RBPs), which serve as versatile regulators of RNA processing.^17^^,^^18^ Beyond their canonical roles in RNA transport, stability, localization, and translation, RBPs specifically coordinate extensive networks of AS events through tissue- and developmental stage-specific combinations of antagonistic RBPs that act on functionally related transcripts.^14^^,^^19^^,^^20^ By recognizing distinct sequence motifs in target pre-mRNAs, splicing RBPs promote or repress the inclusion of exons, thereby modulating the production of alternative splice isoforms.^8^^,^^20^ For example, PTBP1 and RBFOX proteins antagonistically regulate neuron-specific AS during cortical neurogenesis.^21^ PTBP1 is expressed in neural progenitor cells and suppresses inclusion of neuronal exon networks to maintain progenitor identity, whereas RBFOX proteins (RBFOX1/2/3) promote the inclusion of these exons to drive the progenitor-to-neuron fate switch.^21^ PTBP2 coordinates a neuron-specific alternative-splicing program required for axonogenesis.^22^ In addition, PTBP2 binding to SYNGAP1 promotes an unproductive splice isoform subject to nonsense-mediated decay.^23^

Pathogenic variants affecting numerous RBPs have been implicated in human genetic disorders.^24^ We previously reported that biallelic pathogenic variants in the RBP gene *RBM42* cause a syndromic NDD characterized by severe central nervous system malformations, developmental delay, and congenital cardiac defects.^25^ RBM42 is a constituent of the U4/U6·U5 tri-snRNP and participates in the assembly of the pre-B spliceosome complex.^26^^,^^27^ RBM42 directly interacts with hnRNP K,^28^ a nuclear RNA-binding protein encoded by the disease-causative gene for Au-Kline syndrome (AKS).^29^ Importantly, patients with *RBM42* pathogenic variants present phenotypic features highly reminiscent of AKS.^25^ Evolutionary conservation of RBM42 function is supported by studies of its orthologs in lower eukaryotes. FgRBP1 in *Fusarium graminearum* and TgRRM1 in *Toxoplasma gondii* contribute to pre-mRNA splicing.^30^^,^^31^ FgRBP1 functions as a pre-mRNA splicing regulator by binding U2AF23 and modulating splicing of ribosomal protein genes,^30^ while TgRRM1 associates with U4/U6 and U5 snRNPs to facilitate mRNA splicing.^31^ Beyond its role in basal splicing, RBM42 has been implicated in stress-responsive gene regulation. Recent work revealed a dual role for RBM42 in regulating *CDKN1A* splicing and translation during the DNA damage response.^32^ In pancreatic ductal adenocarcinoma, RBM42 promotes translation of oncogenic transcripts by remodeling the *MYC* 5′ UTR.^33^ Despite these findings, the endogenous splicing targets of RBM42, particularly its regulation of neuron-specific isoforms and its role in AS during human brain development, remain uncharacterized. Here, we show that RBM42 is required for cortical development and regulates a conserved splicing program enriched in cytoskeletal genes. Correcting *NIN* exon 18 mis-splicing partially restored cytoskeletal organization and cell proliferation in RBM42-deficient cells.

## Results

### Nestin-Cre-mediated *Rbm42* conditional knockout mice exhibit perinatal lethality and gross developmental abnormalities

To investigate the role of RBM42 in mammalian neurodevelopment, we generated nervous system-specific *Rbm42* conditional knockout offspring by crossing *Rbm42*^flox/flox^ mice with *Rbm42*^flox/+^; Nestin-Cre^+^ mice (Figures S1A–S1D). Nestin-Cre-mediated *Rbm42* cKO offspring were obtained at the expected Mendelian ratio (18/73 or 0.247, Table 1) but died shortly after birth (within ∼12 h postpartum) and presented with pronounced cyanosis (Figure 1A). Immunoblotting of P0.5 cortical lysates showed near-complete depletion of RBM42 protein in cKO mice compared with *Rbm42*^*flox/flox*^ littermate controls, consistent with effective RBM42 protein depletion in the CNS (Figure 1B). PCR genotyping identified the expected Rbm42 and Nestin-Cre products (Figure 1C). By late gestation (embryonic day 18.5, E18.5), *Rbm42* cKO embryos exhibited global growth impairment, as evidenced by a 10.4% reduction in body weight (1.054 ± 0.035 g vs. 1.176 ± 0.016 g in controls; *p* < 0.05) and a 6.5% decrease in crown-rump length (17.61 ± 0.32 mm vs. 18.84 ± 0.37 mm; *p* < 0.05) (Figures 1D–1F). Gross examination of E18.5 *Rbm42* cKO embryos revealed cortical hemorrhagic lesions (Figures 1G and 1H). Histologic analysis further demonstrated severe loss of cortical layering (Figures 2A–2C). To assess vascular organization associated with cortical hemorrhage, we performed CD31 and ZO-1 immunofluorescence staining at E12.5, E14.5, and E18.5. CD31^+^ vascular profile density was unchanged in *Rbm42* cKO dorsal telencephalon at E12.5 but was significantly reduced in cKO cortices by E14.5 (Figures S2A–S2D). At E18.5, severe cortical parenchymal collapse precluded reliable whole-cortex vascular density quantification (Figure S2E). All *Rbm42* cKO neonates examined died within 12 h after birth (18/18; Table 1).

**Table 1:** Genotype distribution and mortality within 12 h after birth

Rbm42 genotype	Nestin-Cre status	Number	Percentage	Group	Lethality
*Rbm42*^flox/flox^	Nestin-Cre^-^	24	32.88%	Control	0/24
*Rbm42*^flox/flox^	Nestin-Cre^+^	18	24.66%	cKO	18/18
*Rbm42*^flox/wt^	Nestin-Cre^-^	15	20.55%	–	0/15
*Rbm42*^flox/wt^	Nestin-Cre^+^	16	21.92%	–	0/16

**Figure 1:**
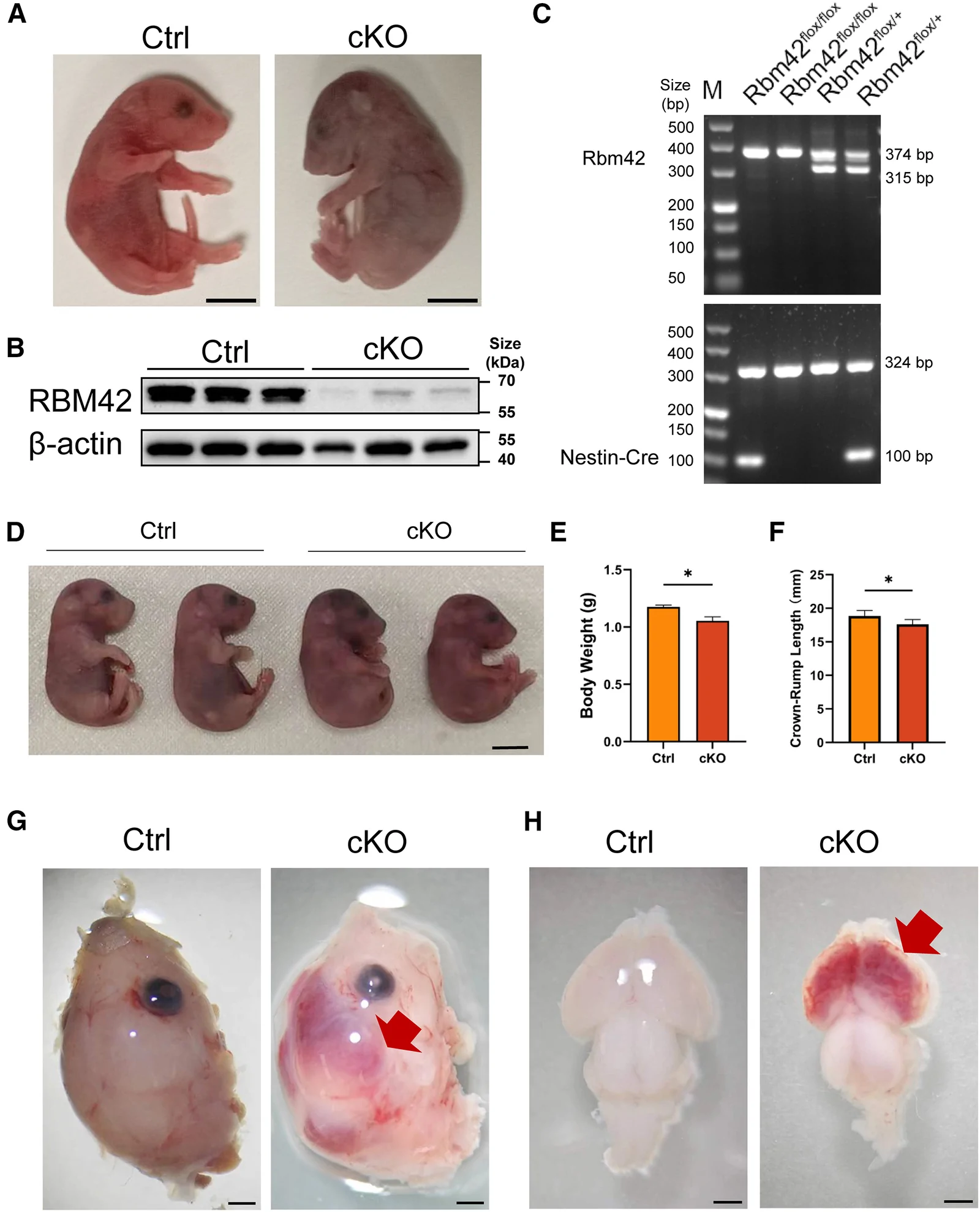
Generation and gross phenotypic characterization of *Rbm42* conditional knockout mice (A) *Rbm42* cKO mice display cyanosis and die within 12 h after birth. Scale bars, 5 mm. (B) Western blot analysis of RBM42 in postnatal day 0.5 (P0.5) Ctrl and *Rbm42* cKO brains; β-actin served as a loading control. (C) PCR genotyping of *Rbm42* alleles and Nestin-Cre transgene. Upper image: *Rbm42* genotyping. The wild-type (wt) allele yields a 315 bp PCR product, whereas the floxed allele yields a 374 bp product. The left two lanes show homozygous floxed (flox/flox) samples, and the right two lanes show heterozygous (wt/flox) samples. Lower image: Nestin-Cre genotyping. The Nestin-Cre transgene yields a PCR product of approximately 100 bp. A 324 bp internal positive control band, specific to the Cre transgenic mouse line, is included to confirm PCR reliability. (D) Representative images of whole-body morphology in control (Ctrl, *Rbm42*^flox/flox^; Nes-Cre^−^) and cKO (*Rbm42*^flox/flox^; Nes-Cre^+^) embryos at E18.5. Scale bars, 5 mm. (E) Body weight. Data are mean ± SEM (*n* = 5 per group). (F) Crown-rump length (CRL). Data are mean ± SEM (*n* = 5 per group). (G) Stereomicroscopy of intact skulls reveals hemorrhagic foci (red arrows) in the cortical region of cKO brains. Scale bars, 1 mm. (H) Stereomicroscopy of brains after skull removal confirming extensive cortical hemorrhage (red arrows) in cKO mice. Scale bars, 1 mm. Statistical significance in (E and F) was assessed using a two-tailed unpaired Student’s *t* test. ∗*p* < 0.05; ∗∗*p* < 0.01; ∗∗∗*p* < 0.001; and ∗∗∗∗*p* < 0.0001.

**Figure 2:**
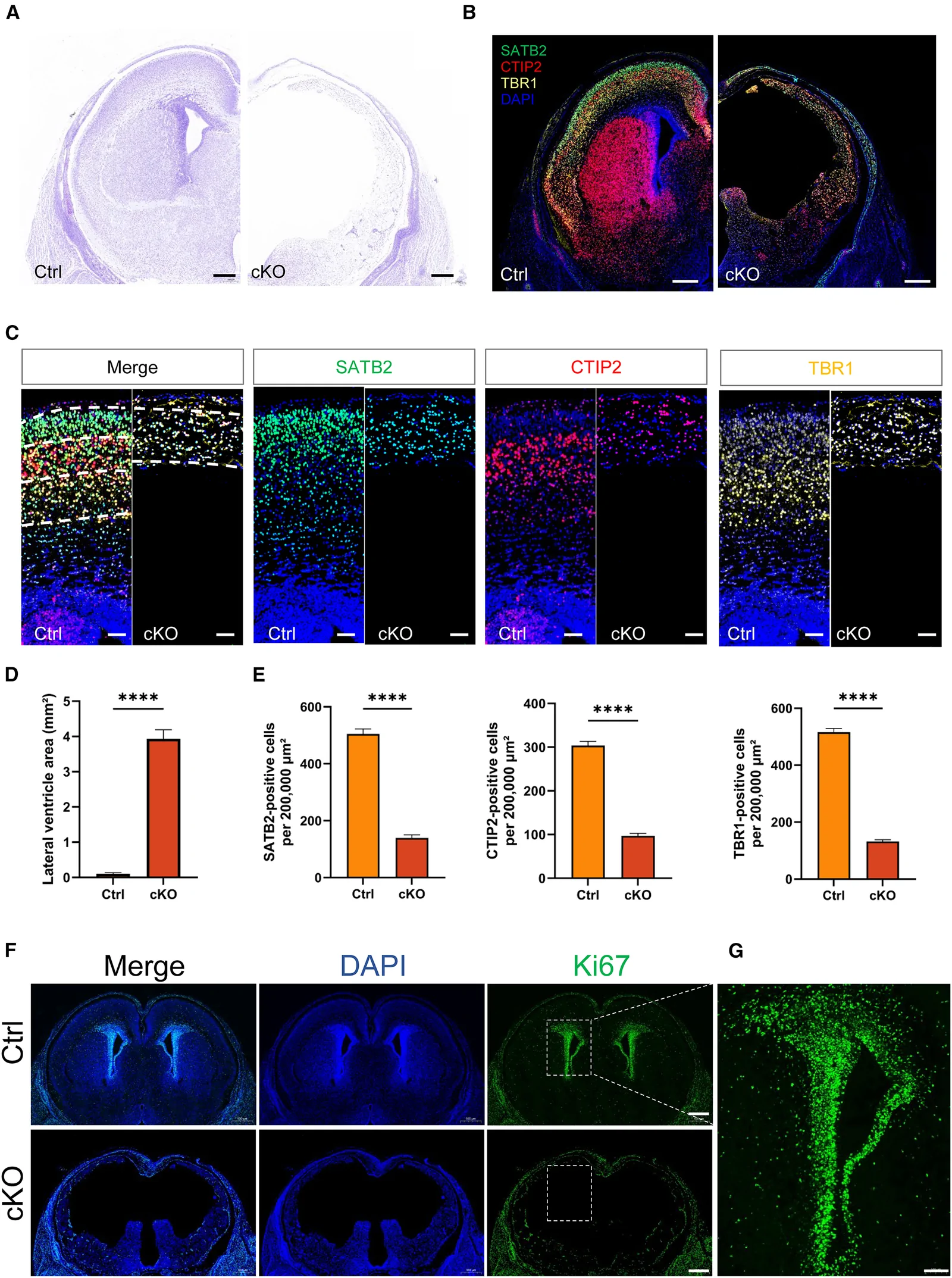
RBM42 loss disrupts cortical lamination and reduces neural progenitor proliferation (A) Nissl-stained coronal sections of E18.5 embryonic heads (with skull) showing reduced cortical thickness and enlarged lateral ventricles in *Rbm42* conditional knockout (cKO) mice compared with control (Ctrl) littermates. Scale bars, 200 μm. (B) Immunofluorescence staining of coronal sections for layer-specific markers SATB2 (upper layers), CTIP2 (deep layers), and TBR1 (deepest layer/subplate) at E18.5. Scale bars, 500 μm. (C) Higher magnification views of the cortical plate from (B). Note impaired laminar organization and intermixing of SATB2^+^, CTIP2^+^, and TBR1^+^ neurons in cKO cortex. Scale bars, 50 μm. (D) Quantification of lateral ventricle area in coronal sections of E18.5 control and *Rbm42* cKO embryos (mean ± SEM; *n* = 5 per genotype). (E) Quantification of cells expressing SATB2, CTIP2, and TBR1 per 200,000 μm^2^ in each section (mean ± SEM; *n* = 5 per genotype). (F) Representative Ki67 immunofluorescence images showing reduced Ki67^+^ proliferative cells in the germinal zones of E18.5 *Rbm42* cKO cortices. Scale bars, 500 μm. (G) 5-fold magnification of Ki67 staining from (F) (Ctrl). Scale bars, 100 μm. Statistical significance in (D and E) was assessed using a two-tailed unpaired Student’s *t* test. ∗*p* < 0.05; ∗∗*p* < 0.01; ∗∗∗*p* < 0.001; and ∗∗∗∗*p* < 0.0001.

### *Rbm42* deletion disrupts neocortical lamination, impairing progenitor proliferation and promoting apoptosis

Histological examination of Nissl-stained coronal sections from *Rbm42* cKO mice revealed severe cortical maldevelopment, manifested as progressively enlarged lateral ventricles and a correspondingly thinned cortical mantle. Significant ventricular expansion was already evident at E14.5 and became more pronounced by E18.5, indicating a progressive defect in cortical development and impaired neurogenesis (Figures 2A, 2D, S3A and S3B). To determine whether *Rbm42* deficiency affects cortical lamination, we examined layer-specific neuron organization in E14.5 and E18.5 cortices. At E14.5, approximately the midpoint of cortical neurogenesis, the numbers of neurons expressing layer-specific markers SATB2, CTIP2, and TBR1 were reduced in *Rbm42* cKO mice compared with those in Ctrl littermates (Figures S3C–S3H). In E18.5 control embryos, neurons expressing the upper-layer marker SATB2 and deep-layer markers CTIP2 and TBR1 were distributed in well-defined, discrete laminar patterns spanning from the cortical plate to the ventricular zone (Figure 2B). In contrast, *Rbm42* cKO cortices exhibited severe disruption of cortical lamination, with SATB2-, CTIP2-, and TBR1-positive neurons interspersed and ectopically clustered in the superficial cortical regions (Figures 2B and 2C). We observed a marked reduction in SATB2^+^, CTIP2^+^, and TBR1^+^ neurons in the cKO cortex compared to controls, with mean counts decreased by 72.4%, 68.1%, and 74.3%, respectively (Figure 2E). These findings suggest that RBM42 is a critical regulator of cortical stratification during neocortical development.

To further investigate the cellular basis of the lamination defect, we assessed neural progenitor proliferation and survival at E18.5. *Rbm42* cKO cortices showed a markedly depleted and disorganized subventricular zone (SVZ) accompanied by a pronounced reduction of Ki67^+^ proliferative cells in the germinal zones (Figure 2F). A higher-magnification image of the Ctrl cortex is shown in Figure 2G to illustrate the Ki67 staining pattern near the ventricular zone. TUNEL assays further demonstrated widespread apoptosis throughout the mutant cortex (Figures S5A and S5B). To determine whether progenitor defects were already evident during early cortical neurogenesis, we examined mitotic progenitors in E12.5 embryonic cortices by phospho-histone H3 (PH3) immunostaining. PH3-positive mitotic cells lining the ventricular surface were significantly reduced in Rbm42 cKO cortices, indicating impaired progenitor mitotic activity at an early neurogenic stage (Figure S4). We next examined the cellular distribution of apoptotic cells by TUNEL/PAX6/βIII-tubulin immunofluorescence staining on E14.5 cortical sections. TUNEL-positive nuclei co-labeled with PAX6 were readily detected in Rbm42 cKO cortices, indicating involvement of the PAX6-positive progenitor/radial glial compartment (Figures S5C and S5D). In addition, many apoptotic cells were distributed within βIII-tubulin-rich cortical regions (Figure S5D), suggesting that both progenitor and neuronal compartments are affected. Together, these findings establish that RBM42 is required for progenitor maintenance and cortical cell survival, and that its loss perturbs cortical lamination by disrupting neurogenesis and promoting cell death.

### RBM42 regulates the splicing of RNAs involved in neurodevelopment and associates with spliceosomal factors

The impaired lamination in *Rbm42* cKO cortical development prompted us to investigate whether RBM42 might regulate neurodevelopmental processes such as neurite outgrowth or cellular proliferation. For these studies, we first generated negative control (shNC) and RBM42-knockdown (shRBM42) stable SH-SY5Y neuroblastoma cell lines via lentiviral transduction of short hairpin RNA. Then, we transfected the shRBM42 cell line with either an empty vector (KD-Vector) or an RBM42 overexpression plasmid (KD-RBM42). Knockdown (KD) efficiency and successful rescue of RBM42 expression were validated at both the transcript and protein levels (Figures 3A–3C, S6A and S6B). RBM42-depleted cells showed reduced proliferative activity, with a lower Ki67-positive fraction than shNC cells; RBM42 re-expression increased the Ki67-positive fraction toward the control level (Figures 3D and 3E). EdU incorporation was also reduced after RBM42 depletion (Figures S6C and S6D). Morphologically, RBM42-deficient SH-SY5Y cells displayed impaired neurite outgrowth (Figure S6E). To further assess the morphological changes induced by RBM42 knockdown in SH-SY5Y cells, we employed label-free imaging and observed that control SH-SY5Y cells exhibited long, thin neurite extensions with complex branching, whereas RBM42-deficient cells displayed markedly shorter neurites, consistent with an impaired neurite outgrowth phenotype (Figures 3F and 3G). To delineate RBM42-dependent splicing alterations, we performed replicate Multivariate Analysis of Transcript Splicing (rMATS) analysis in the shRBM42 cells. Splicing analysis identified 640 significant AS events corresponding to 526 distinct genes that were differentially regulated in shRBM42 cells relative to shNC controls (false discovery rate (FDR) < 0.05, |IncLevelDifference| > 0.1; Figure 4A and Table S2). Exon skipping was the predominant splicing event, representing 77.5% (496 of 640) of all alterations (Figure 4A and Table S2). To further characterize the structural and sequence features of RBM42-regulated cassette exons, we analyzed exon-intron architecture and splice-site strength of significant skipped exon (SE) events identified by rMATS. RBM42-regulated cassette exons displayed a median length of 86 nt (interquartile range [IQR]: 46.75–134 nt). The median upstream and downstream intron lengths were 2,052 nt (IQR: 685.25–6,217 nt) and 2,136.5 nt (IQR: 866.5–4,970 nt), respectively. To evaluate splice-site strength, MaxEnt scores were calculated for both donor and acceptor sites.^34^ The median 5′ splice site (donor) score was 8.31 (IQR: 6.39–9.45), whereas the median 3′ splice site (acceptor) score was 7.25 (IQR: 5.05–9.32) (Tables 2 and S2A).

**Figure 3:**
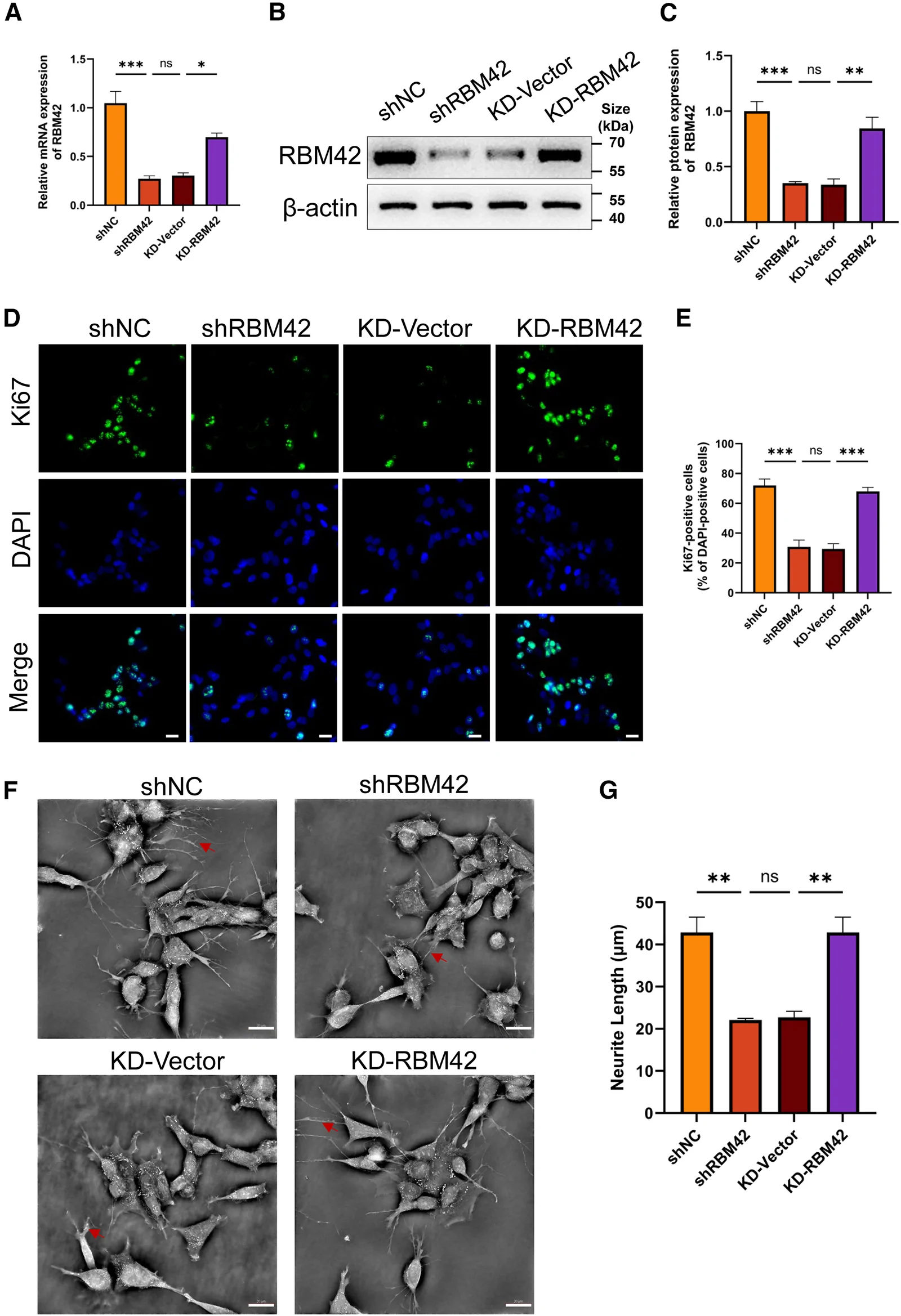
RBM42 is required for SH-SY5Y proliferation and neurite growth (A) Relative expression of *RBM42* mRNA using RT-qPCR in SH-SY5Y stable cell lines. shNC, shRBM42, shRBM42 + vector plasmid (KD-Vector), and shRBM42 + RBM42 plasmid (KD-RBM42) are grouped. Data are presented as mean ± SEM from three independent biological experiments (*n* = 3). (B) Western blot of RBM42 protein levels in SH-SY5Y stable cell lines; β-actin served as a loading control. (C) Statistical analysis of RBM42 protein expression levels quantified by grayscale values. Data are presented as mean ± SEM from three independent biological experiments (*n* = 3). (D) Representative images of Ki67 immunofluorescence staining to assess proliferative activity in SH-SY5Y cells. Scale bars, 20 μm. (E) Quantification of Ki67-positive cells expressed as a percentage of total DAPI-stained nuclei. For each independent experiment, three randomly selected fields were analyzed per condition. Statistical analysis was performed using biological replicate means (*n* = 3). Data are presented as mean ± SEM. (F) Representative intensity diffraction tomography images of shNC, shRBM42, KD-Vector, and KD-RBM42 SH-SY5Y cell lines. Red arrows indicate extended neurites. Scale bars, 20 μm. (G) Quantification of neurite lengths for shNC, shRBM42, KD-Vector, KD-RBM42 SH-SY5Y cell lines. For each independent experiment, three randomly selected fields were analyzed per condition; field-level neurite measurements were averaged to generate one biological-replicate mean for statistical inference (*n* = 3). Data are presented as mean ± SEM. KD, knockdown. Statistical significance in (A, C, E, and G) was assessed using ordinary one-way ANOVA followed by Tukey’s multiple-comparisons test. ∗*p* < 0.05; ∗∗*p* < 0.01; and ∗∗∗*p* < 0.001. ns, not significant.

**Figure 4:**
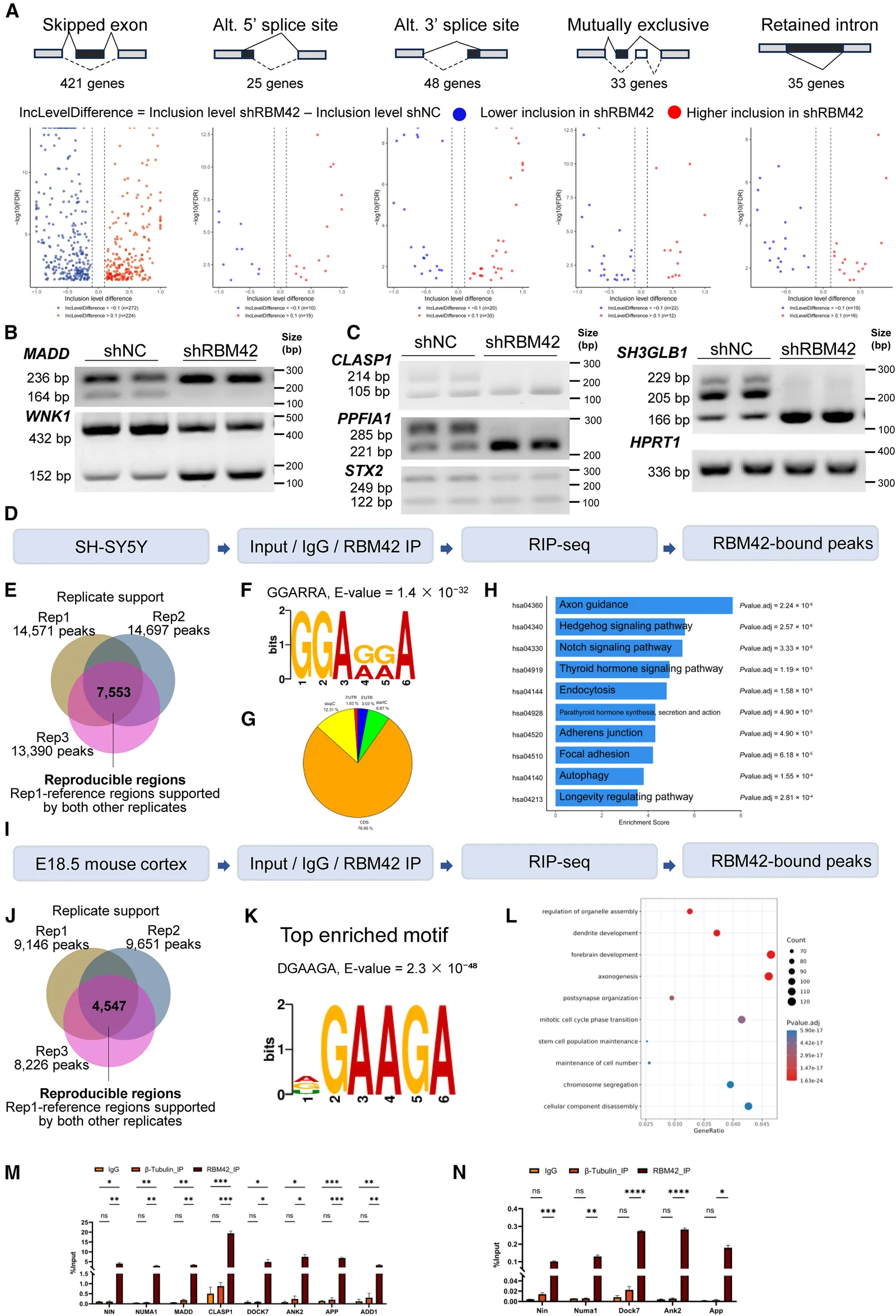
Transcriptomic profiling of RBM42 splicing-regulated and RNA-binding targets in SH-SY5Y cells and embryonic mouse cortex (A) (Top) Types of alternative splicing (AS) events, and number of genes having at least one AS event for each type in shRBM42 vs. shNC SH-SY5Y cells. IncLevelDifference was calculated as the mean inclusion level in shRBM42 samples minus that in shNC samples. Positive values indicate higher inclusion in shRBM42 cells, whereas negative values indicate lower inclusion. (Bottom) Volcano plots were generated from *n* = 2 independent biological replicates per group (Table S2). KD, knockdown; Alt. Exon, alternative exon. The vertical axis of the volcano plots shows −log_10_(FDR), where FDR denotes the false discovery rate. (B) RT-PCR validation of alternatively spliced skipped exons (SEs) in OMIM-listed human disease genes. *HPRT1* served as a loading control. (C) RT-PCR validation of SEs in additional OMIM-listed genes. (D) Schematic workflow of RBM42 RIP-seq using SH-SY5Y cells. Input, IgG control, and anti-RBM42 immunoprecipitation samples were included. (E) Schematic summary of RIP-seq replicate support in SH-SY5Y cells. Numbers outside the circles indicate total peaks per replicate; the central number denotes reproducible regions defined using Rep1 as the coordinate reference and supported by both other replicates (Table S3). (F) Top sequence motifs enriched in RBM42-binding peaks identified by DREME motif discovery. (G) Genomic distribution of reproducible RBM42-associated regions across 5′ untranslated regions (5′ UTRs), start-codon regions (startC), coding sequences (CDSs), stop-codon regions (stopC), and 3′ untranslated regions (3′ UTRs). The startC and stopC regions extend 100 bp upstream and downstream of the respective codon. (H) KEGG pathway analysis of RBM42 target transcripts, highlighting significantly enriched pathways. *p* values were calculated by Fisher’s exact test and adjusted by the Benjamini-Hochberg method. Adjusted *p* values are indicated in the figure. (I) Schematic workflow of RBM42 RIP-seq using E18.5 mouse cortical lysates. Input, IgG control, and anti-RBM42 immunoprecipitation samples were included. (J) Schematic summary of RIP-seq replicate support in E18.5 mouse cortical tissues. Numbers outside the circles indicate total peaks per replicate; the central number denotes reproducible regions defined using Rep1 as the coordinate reference and supported by both other replicates (Table S8). (K) Top enriched motif identified by DREME analysis of RBM42-bound regions. (L) GO biological process enrichment analysis of genes associated with reproducible RBM42-bound regions identified from E18.5 mouse cortical RIP-seq. Dot color indicates the adjusted *p* value, and dot size indicates the number of genes associated with each GO term. (M) RIP-qPCR validation of selected neurodevelopmental and cytoskeleton-associated candidate transcripts in SH-SY5Y cells. Candidate transcripts were selected based on RIP-seq enrichment, RBM42-dependent alternative splicing, and functional relevance to neurodevelopmental or cytoskeletal regulation. IgG and β-tubulin immunoprecipitations were used as negative controls. Data are mean ± SEM from three independent biological replicates. (N) RIP-qPCR validation of selected candidate transcripts in E18.5 mouse cortical tissue. IgG and β-tubulin immunoprecipitations were used as negative controls. Data are mean ± SEM from three independent biological replicates. Statistical significance was assessed using two-way ANOVA with Tukey’s multiple-comparisons correction. ns, not significant; ∗*p* < 0.05, ∗∗*p* < 0.01, ∗∗∗*p* < 0.001, and ∗∗∗∗*p* < 0.0001.

**Table 2:** Structural characteristics and splice-site strength of RBM42-regulated cassette exons identified by rMATS

Metric	Median	Q1	Q3
Exon length (nt)	86.00	46.75	134.00
Upstream intron length (nt)	2052.00	685.25	6217.00
Downstream intron length (nt)	2136.50	866.50	4970.00
5′ splice site MaxEnt (donor)	8.31	6.39	9.45
3′ splice site MaxEnt (acceptor)	7.25	5.05	9.32

Values are shown as median (IQR; Q1–Q3) for significant skipped exon (SE) events (*n* = 496) identified by rMATS in shRBM42 versus shNC. Upstream and downstream intron lengths are defined relative to the transcriptional direction. Splice-site strength was evaluated using MaxEnt scores for 5′ donor (MaxEnt5) and 3′ acceptor (MaxEnt3) sites. Complete event-level data are provided in Table S2A.

To elucidate how RBM42 interfaces with the splicing machinery, we profiled proteins associated with RBM42 by co-immunoprecipitation followed by mass spectrometry (Co-IP-MS) in SH-SY5Y cells (Figure S7A). Nuclear extracts were used to enrich for spliceosomal interactions, and extract purity was verified with nuclear marker proteins (Figure S7B). Co-immunoprecipitation of endogenous RBM42 (with IgG as a control) identified 112 proteins in the RBM42 IP group versus 43 in the IgG control. The exploratory IP-MS analysis identified 81 proteins detected in the RBM42 IP but not in the IgG control, comprising RBM42 itself and 80 non-bait candidate associated proteins (Figures S7A–S7C and Table S7C). Notably, among these RBM42-associated proteins were key splicing regulators, including the U5 snRNP component EFTUD2 and heterogeneous nuclear ribonucleoprotein K (hnRNP K) (Figures S7D and S7G). The detection of these splicing-related proteins is consistent with the known association of RBM42 with spliceosomal complexes. Pathway enrichment analysis of the candidate RBM42-associated proteins revealed a strong over-representation of factors involved in RNA processing, particularly mRNA splicing (Figures S7E and S7F). Among the alternatively spliced transcripts, multiple NDD-associated genes were identified as RBM42-related targets, including *MADD* (OMIM: 603584) and *WNK1* (OMIM: 605232), which modulate neurotransmitter release and ion transport, respectively (Figure 4B and Table S2F). RBM42 loss increased inclusion of exon 34 in *MADD* and caused skipping of exon 12 in *WNK1*. Additional splicing alterations were observed in genes associated with the structures and functions of neural cells, including skipping of exon 23 in the microtubule-stabilizing factor *CLASP1* (within the microtubule-binding region), exon 24 in the synaptic scaffolding protein *PPFIA1* (within the SAM 2 domain), exon 10 in the vesicle trafficking component *STX2* (adjacent to the t-SNARE coiled-coil homology domain), and exon 7 in the endocytic regulator *SH3GLB1* (within the BAR domain) (Figure 4C and Table S2F). Densitometric quantification of the RT-PCR bands is provided in Figures S8D and S8E.

### RBM42 binds and regulates neurodevelopmental and cytoskeleton-associated transcripts

To determine which endogenous RNAs are likely to be bound targets of RBM42, we performed RNA immunoprecipitation followed by high-throughput sequencing (RIP-seq). RBM42 RIP-seq identified 7,553 RBM42-binding peaks reproducibly detected across all three biological replicates, mapping to 2,730 transcript IDs and 2,099 unique gene symbols (Figures 4D and 4E and Table S3). Motif analysis revealed that RBM42-binding sites are strongly enriched for GGA-containing sequences, including a bipartite GGA motif (Figure 4F). Mapping of RBM42 peaks relative to gene features showed that >70% of binding sites reside in coding sequences (CDS), with ∼3% and ∼1% of peaks in 5′ and 3′ untranslated regions, respectively (Figure 4G). KEGG pathway analysis revealed significant enrichment of neurodevelopment-related signaling pathways, such as axon guidance, Hedgehog signaling, and Notch signaling (Figure 4H). GO analysis indicated broad involvement in neurobiological processes and structures (Figures S8A–S8C). Then, we performed RBM42 RIP-seq using E18.5 mouse cortical tissues (Figure 4I). Across three biological replicates, 4,547 reproducible RBM42-bound regions were identified, mapping to 2,943 transcript IDs and 2,698 unique gene symbols (Figure 4J and Table S8). DREME motif analysis identified a GAAGA-centered motif as the top enriched sequence element (DGAAGA; *E* value = 2.3 × 10^−48^), consistent with the G/A-rich motifs detected in SH-SY5Y RIP-seq (Figure 4K). GO biological process analysis revealed enrichment of RBM42-bound transcripts in neurodevelopmental processes, including dendrite development, forebrain development, axonogenesis, and postsynaptic organization (Figure 4L). RIP-qPCR further confirmed RBM42 enrichment on selected neurodevelopmental and cytoskeleton-associated candidate transcripts in both SH-SY5Y cells and E18.5 mouse cortical tissues, using IgG and β-tubulin IPs as negative controls (Figures 4M and 4N). These results establish an *in vivo* RBM42 RNA-binding landscape in embryonic mouse cortex and support the physiological relevance of the RBM42-associated transcript network identified in SH-SY5Y cells.

To identify candidate RBM42-associated splicing targets, we overlapped RBM42-bound genes identified by RIP-seq with genes showing alternative splicing after RBM42 depletion in SH-SY5Y cells, yielding 113 candidate genes (Figure 5A and Table S4). These transcripts, which are bound by RBM42 and exhibit altered splicing upon RBM42 loss, are most significantly enriched in the cell-cortex cytoskeleton organization pathway (Figure 5B). Among these SH-SY5Y candidates, we prioritized *NUMA1* for further analysis as a cytoskeleton-associated RBM42-regulated splicing target (Figures 5A–5C). To extend our findings *in vivo*, we performed RNA-seq on brain tissue from *Rbm42* cKO mice. The rMATS analysis identified 1,158 significantly altered splicing events affecting 878 genes (Figure S9A and Table S5), including key neurodevelopmental regulators such as *Dock7*, *App*, *Wnk1*, and *Sh3glb1* (Figures S9B and S9C). We then compared splicing changes in *Rbm42* cKO mouse brains with those in RBM42-knockdown human cells, identifying 87 orthologous genes with AS changes in both datasets (Figure 5D and Table S6). These conserved AS-regulated genes were highly enriched in cytoskeletal pathways, indicating a shared mechanistic axis across species. Consistent with this, GO analysis identified microtubule binding and tubulin binding as the top molecular functions, prominent enrichment of biological processes related to microtubule polymerization and depolymerization, and cellular components associated with cell-substrate junctions, the cell cortex, microtubules, growth cones, and distal axons (Figures 5E, S10A and S10B). Among these conserved AS-regulated genes, *NIN*/*Nin* was prioritized because it showed significant AS changes in both the human and mouse datasets (Figure 5D), and mouse *Nin* was further identified in the overlap between RBM42-bound transcripts from mouse cortical RIP-seq and significant AS genes in *Rbm42* cKO cortices (Tables S5 and S8). Thus, *NIN*/*Nin* and *NUMA1*/*Numa1* were selected as complementary RBM42-associated cytoskeletal splicing targets for subsequent validation (Figure 5C).

**Figure 5:**
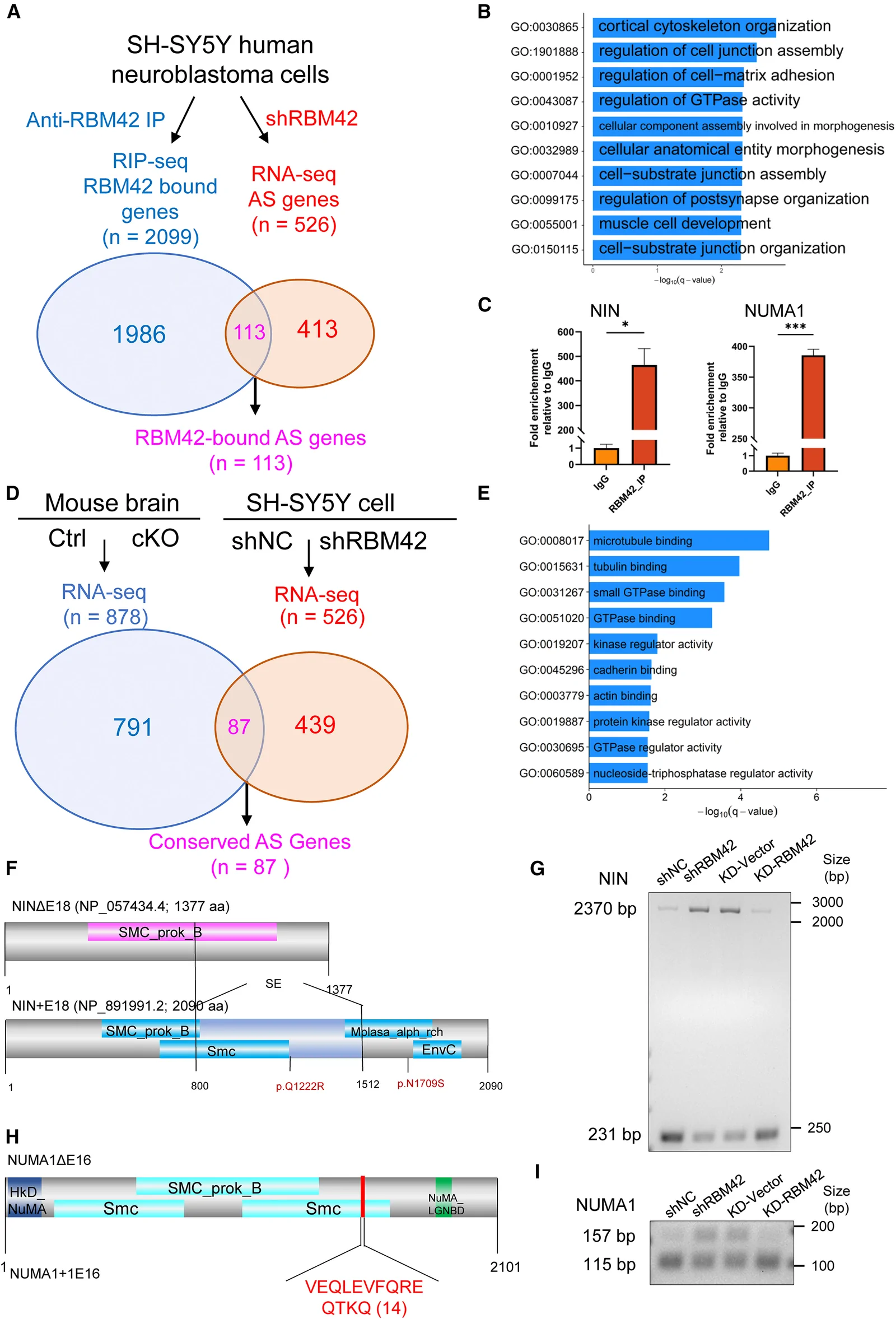
Integrated analysis of RBM42-dependent alternative splicing and RNA-associated targets in human and mouse neural systems (A) Venn diagram showing the gene-level intersection between genes with at least one significant RBM42-dependent AS event and protein-coding genes whose transcripts contain reproducible exon-overlapping RBM42 RIP-seq peaks (Table S4); values indicate numbers of genes, and 113 genes are shared. (B) Gene Ontology (GO) biological process enrichment for the candidate genes identified in (A). The top ten significantly enriched terms are shown. The horizontal axis shows −log10(q value). (C) RIP-qPCR validation of RBM42 binding to *NUMA1* and *NIN* transcripts. Data show enrichment in RBM42 IP relative to IgG control IP and are presented as mean ± SEM from three independent biological replicates (*n* = 3). (D) Venn diagram shows orthologous genes with at least one significant AS event in SH-SY5Y cells and mouse brain; values indicate numbers of genes, and 87 genes are shared (n indicates number of genes; see Table S6). Here, “conserved” indicates gene-level evidence of significant alternative splicing in both species. (E) GO molecular function enrichment for orthologous genes showing AS changes in both datasets from (D); top ten terms shown. The horizontal axis shows −log10(q value). (F) Protein models are labeled NINΔE18 and NIN+E18. The 713-amino-acid sequence present in NIN+E18 is shown in light purple. A CDD-predicted SMC_prok_B superfamily match overlaps part of this sequence. (G) RT-PCR validation shows a higher relative abundance of the NIN exon 18-included product after RBM42 depletion and a shift toward shNC levels after RBM42 re-expression. (H) Models are labeled NUMA1ΔE16 and NUMA1+E16. The 14-amino-acid sequence present in NUMA1+E16 is shown in red, and predicted domain matches are shown in cyan; a predicted Smc-superfamily match overlaps the inserted sequence. (I) RT-PCR validation shows a higher relative abundance of the NUMA1 exon 16-included product after RBM42 depletion and a shift toward shNC levels after RBM42 re-expression. Statistical significance in (C) was assessed using unpaired two-tailed Student’s *t* tests. ∗*p* < 0.05 and ∗∗∗*p* < 0.001. HkD_NuMA, Hook domain of nuclear mitotic apparatus protein (NuMA) (NCBI CDD: cd22224); NuMA_LGNBD, LGN-binding domain of NuMA (NCBI CDD: cd22298); SMC_prok_B, chromosome segregation protein SMC, bacterial type (NCBI CDD: cl37069); Smc, chromosome segregation ATPase Smc (NCBI CDD: cl34174); Mplasa_alph_rch, helix-rich Mycoplasma protein (NCBI CDD: cl37461); EnvC, septal ring factor EnvC (NCBI CDD: cl34844).

We next examined two RBM42-regulated genes that showed AS changes in both human cells and mouse brains. In human SH-SY5Y cells, RT-PCR showed that RBM42 depletion increased inclusion of *NIN* exon 18 (GenBank: NM_182946.2) and *NUMA1* exon 16 (GenBank: NM_006185.4), and these splicing changes were restored by RBM42 re-expression (Figures 5G–5I). In *Rbm42* cKO mouse brains, RT-PCR likewise confirmed increased inclusion of *Nin* exon 16 (GenBank: NM_001081453.1) and *Numa1* exon 15 (GenBank: NM_001403544.1), providing independent evidence of concordant exon-inclusion changes in these selected genes *in vivo* (Figures S9E and S9G). We next examined these targets at the protein level in embryonic cortices from control and *Rbm42* cKO mice. For *Nin*, both the exon 16–included (NIN+E16) and exon 16–skipped (NINΔE16) isoforms were reduced in *Rbm42* cKO cortices, whereas the NIN+E16/NINΔE16 ratio was increased (Figure S9H). This ratio is consistent with the RNA-splicing shift shown in Figure S9E. For *Numa1*, altered splicing of *Numa1* exon 15 was confirmed by RT-PCR, but the ∼2 kDa size difference between predicted isoforms precluded reliable resolution by western blotting; total NUMA1 protein abundance remained unchanged (Figure S9H). Together, *Nin* dysregulation was supported at the RNA and protein levels, whereas *Numa1* dysregulation was confirmed at the RNA-splicing level. CDD analysis returned SMC-superfamily sequence matches overlapping the exon-derived peptide segments. Specifically, the peptide encoded by *NIN* exon 18 overlaps a predicted coiled-coil region and an SMC-superfamily sequence match (Figures 5F and S9D), whereas the peptide encoded by *NUMA1* exon 16 overlaps similarly predicted features (Figures 5H and S9F). SMC-family proteins have been linked to microtubule stability and genome integrity.^35^ However, these in silico predictions do not establish SMC-family biochemical function in NIN or NUMA1. These isoform changes may influence their cytoskeletal functions, although this possibility requires direct functional validation. NUMA1 regulates microtubule organization in axonal growth cones and is required for normal axonal growth,^36^ while candidate variants in NIN, including p.Gln1222Arg (p.Q1222R) in exon 18, were reported in two sisters with microcephalic primordial dwarfism.^37^ As an exploratory comparison with a clinically curated gene set, we tested whether the 87 conserved RBM42-dependent AS genes were enriched for neurodevelopmental DDG2P genes. Of the 79 conserved targets included in the analysis, 14 overlapped this gene set, representing significant enrichment (odds ratio = 2.07; two-sided Fisher’s exact test, *p* = 0.0190). To provide a term-level functional interpretation, we additionally summarized enriched GO Biological Process terms using semantic reduction (rrvgo) and highlighted neurodevelopment-related processes (e.g., neuronal morphogenesis, axon development, neurotransmitter transport and secretion, the synaptic vesicle cycle, and synapse organization) in a summary plot (Figure S10C). Taken together, these findings suggest that RBM42 regulates a cross-species set of AS-regulated genes involved in cytoskeletal organization and neural development, with selected isoform changes potentially affecting cytoskeletal protein domains.

### RBM42 deficiency disrupts cytoskeletal architecture during cortical development

Given the strong enrichment of cytoskeletal genes among RBM42’s splicing targets, we next asked whether RBM42 loss leads to defects in cytoskeletal structures during neurodevelopment. In *Rbm42* cKO embryos at E18.5, immunofluorescence for βIII-tubulin, an early neuronal-lineage marker, revealed a profound disorganization of βIII-tubulin-labeled neuronal processes and microtubule-rich neuronal structures in the cortex. Radial glial fibers were fragmented and aberrantly branched in the cKO cortex. Cortical neurons showed disrupted polarity, with neurites projecting in irregular trajectories (Figure 6A). As early as E14.5, βIII-tubulin expression was attenuated and displayed a sparser distribution throughout the cortical layers of the cKO cortex (Figure 6B). Vimentin staining, which marks intermediate filaments in radial glia, revealed region-specific defects in *Rbm42* cKO embryos. The radial glial scaffold within the ventricular zone appeared largely preserved, yet the superficial cortical layers showed markedly reduced vimentin signals, consistent with disruption of radial glial fiber organization in the upper cortex (Figure 6A). These *in vivo* observations demonstrate that RBM42 is required for proper assembly and maintenance of neuronal and radial glial cytoskeletal architecture during cortical development. Consistent with the observed cytoskeletal phenotypes, exploratory KEGG analysis of the RBM42-associated proteins identified enrichment of the adherens junction and amyotrophic lateral sclerosis pathways, suggesting that the RBM42-associated protein network may intersect with cytoskeletal organization and neurologically relevant processes (Figure S7E). In line with this, phalloidin staining of F-actin in SH-SY5Y cells revealed that RBM42-deficient cells adopted an enlarged, spread morphology with thick actin filament bundles concentrated at the cell periphery (Figures 6C and 6D). This dysregulation of cytoskeletal networks underscores RBM42’s critical role in actin cytoskeletal organization and the maintenance of neuronal cell morphology.

**Figure 6:**
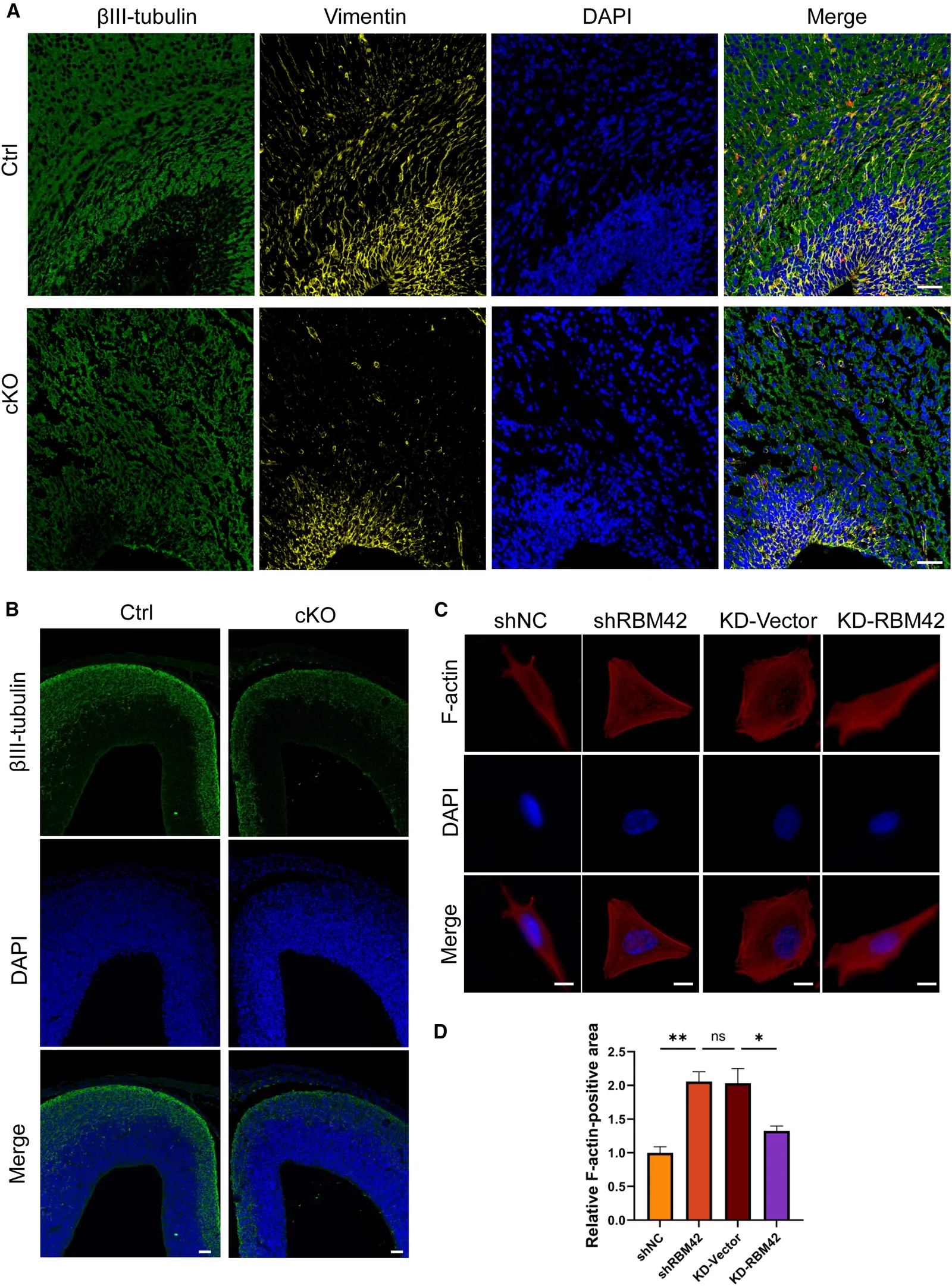
RBM42 deficiency disrupts cytoskeletal organization (A) Immunofluorescence of βIII-tubulin (microtubules) and vimentin (intermediate filaments) in coronal brain sections from E18.5 *Rbm42* conditional knockout (cKO) and control (Ctrl) mice, showing disorganization and altered distribution of cytoskeletal components in cKO compared with Ctrl littermates. Representative images from 5 Ctrl and 5 cKO embryos are shown. Scale bars, 40 μm. (B) Immunofluorescence of βIII-tubulin (microtubules) in coronal brain sections from E14.5 *Rbm42* cKO and Ctrl mice. Representative images from 5 Ctrl and 7 cKO embryos are shown. Scale bars, 50 μm. (C) Immunofluorescence of phalloidin (F-actin, red) in shNC, shRBM42, KD-Vector, and KD-RBM42 SH-SY5Y cells. Scale bars, 10 μm. (D) Quantification of the relative F-actin-positive area corresponding to (C), normalized to the mean of the shNC group. Data are presented as mean ± SEM from three independent experiments. Statistical significance was assessed using ordinary one-way ANOVA followed by Tukey’s multiple-comparisons test. ∗*p* < 0.05 and ∗∗*p* < 0.01; ns, not significant.

### Correction of *NIN* exon 18 mis-splicing partially ameliorates cytoskeletal and proliferative defects in RBM42-deficient cells

Conserved Domain Database analysis identified a predicted SMC-related sequence match overlapping the 713-amino-acid segment present in NIN+E18 (Figure 5F). We therefore tested whether increased *NIN* exon 18 inclusion contributes to the cytoskeletal phenotype caused by RBM42 depletion. RBM42-deficient SH-SY5Y cells were treated with a splice-switching antisense oligonucleotide (ASO) designed to block *NIN* exon 18 inclusion. RT-PCR analysis confirmed that RBM42 knockdown markedly increased *NIN* exon 18 inclusion and that control ASO treatment had little effect (Figures 7A and 7B). In contrast, *NIN* ASO treatment significantly reduced exon 18 inclusion and shifted the *NIN* splicing pattern toward that observed in shNC cells (Figures 7A and 7B). We next assessed the βIII-tubulin cytoskeletal phenotype. RBM42-deficient cells exhibited an increased βIII-tubulin-positive area normalized to total cell area, and this abnormality persisted after control ASO treatment (Figures 7D and 7E). *NIN* ASO treatment partially normalized this phenotype, as shown by a significant reduction in the normalized βIII-tubulin-positive area relative to control ASO-treated RBM42-deficient cells (Figures 7D and 7E). As a complementary isoform-expression approach, we expressed the exon 18-skipped NINΔE18 isoform or the exon 18-included NIN+E18 isoform in RBM42-deficient SH-SY5Y cells (Figure 7C). Expression of NINΔE18 partially ameliorated the abnormal βIII-tubulin phenotype, whereas NIN+E18 did not produce a significant rescue (Figures 7F and 7G). Direct comparison further showed that the effects of NINΔE18 and NIN+E18 on the βIII-tubulin phenotype were significantly different, supporting an exon 18-dependent functional difference between the two NIN isoforms. Together with the splice-switching results, these data support an isoform-dependent contribution of *NIN* splicing to the βIII-tubulin cytoskeletal phenotype. In parallel, a splice-switching ASO targeting *NUMA1* exon 16 effectively reduced aberrant exon 16 inclusion but did not significantly normalize the βIII-tubulin-positive area under the same assay conditions (Figures S11A–S11D).

**Figure 7:**
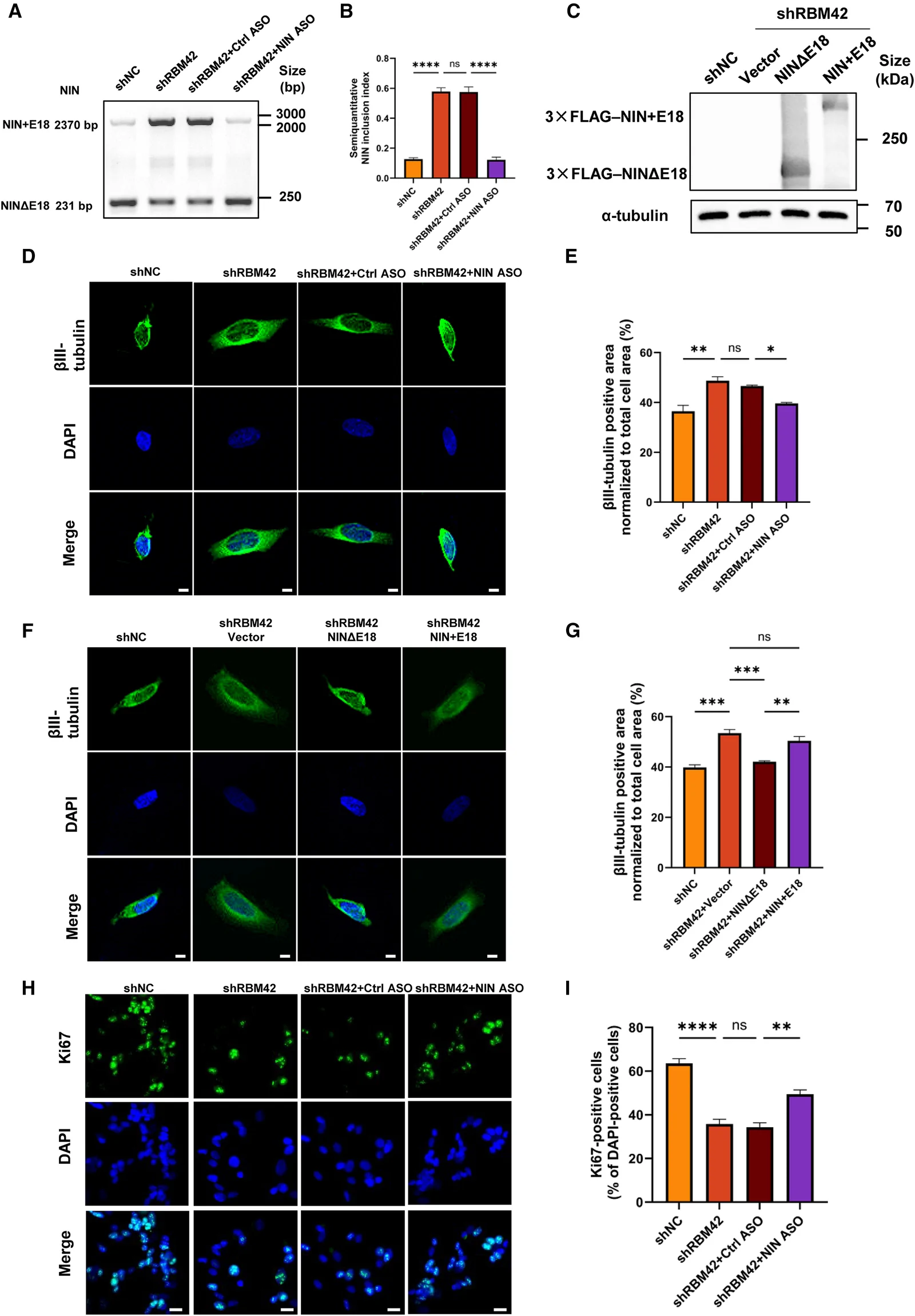
Functional validation of RBM42-dependent *NIN* splicing in SH-SY5Y cells (A) RBM42 depletion was associated with a higher semiquantitative *NIN* exon 18-inclusion index, whereas the *NIN*-targeting ASO shifted the splicing pattern toward exon 18 skipping. (B) Semiquantitative *NIN* exon 18 inclusion index in the indicated groups. Data are presented as mean ± SEM from three independent experiments. (C) Immunoblot analysis using an anti-FLAG antibody confirming expression of the indicated 3×FLAG-tagged NIN isoforms in SH-SY5Y cells. α-tubulin was used as a loading control. (D) Representative immunofluorescence images of βIII-tubulin staining in SH-SY5Y cells under the indicated conditions. *NIN*-targeting ASO treatment partially ameliorated the abnormal βIII-tubulin phenotype induced by RBM42 depletion. Scale bars, 2 μm. (E) Quantification of βIII-tubulin-positive area normalized to total cell area in the indicated groups. Data are presented as mean ± SEM from three independent experiments. For each independent experiment, 5–20 randomly selected fields were analyzed per condition. (F) Representative immunofluorescence images of βIII-tubulin staining in SH-SY5Y cells transduced with shNC or shRBM42 together with the indicated NIN isoform constructs. Expression of NINΔE18 resulted in a significantly greater restoration of βIII-tubulin organization than expression of NIN+E18, supporting a differential effect of the two NIN isoforms. Scale bars, 2 μm. (G) Quantification of βIII-tubulin-positive area normalized to total cell area in the indicated groups. Data are presented as mean ± SEM from three independent experiments. (H) Representative immunofluorescence images of Ki67 staining in SH-SY5Y cells under the indicated conditions. Nuclei were counterstained with DAPI. *NIN*-targeting ASO treatment partially ameliorated the reduction in Ki67-positive cells induced by RBM42 depletion. Scale bars, 20 μm. (I) Quantification of the proportion of Ki67-positive cells among DAPI-positive cells in the indicated groups. For each independent experiment, three randomly selected fields were analyzed per condition. Data are presented as mean ± SEM from three independent experiments. Statistical analyses in (B, E, G, and I) were performed using ordinary one-way ANOVA followed by Tukey’s multiple comparisons test. ns, not significant; ∗*p* < 0.05; ∗∗*p* < 0.01; ∗∗∗*p* < 0.001; and ∗∗∗∗*p* < 0.0001.

Because *Rbm42* cKO cortices showed reduced progenitor proliferation and increased TUNEL positivity, we next assessed the corresponding cellular phenotypes under the ASO rescue conditions. Ki67 immunostaining showed that RBM42 depletion reduced the proportion of Ki67-positive SH-SY5Y cells, and this reduction was not altered by control ASO treatment. *NIN* ASO treatment significantly increased the Ki67-positive fraction relative to control ASO-treated RBM42-deficient cells, although the mean remained below that observed in shNC cells (Figures 7H and 7I). In contrast, *NUMA1* ASO did not significantly increase the Ki67-positive fraction (Figures S11E and S11F). RBM42 depletion also increased the proportion of TUNEL-positive cells. However, neither *NIN* nor *NUMA1* ASO significantly reduced the TUNEL-positive fraction under the conditions tested (Figures S12A–S12D). These results indicate that correction of NIN exon 18 mis-splicing partially ameliorates the reduced proliferative fraction, whereas correction of either individual splicing event is insufficient to rescue the TUNEL phenotype. Overall, the ASO and isoform-expression experiments support a phenotype-selective contribution of *NIN* exon 18 mis-splicing to the βIII-tubulin cytoskeletal abnormality and reduced proliferative capacity associated with RBM42 depletion. These findings are consistent with *NIN* representing one functionally relevant component of the broader RBM42-regulated splicing network implicated in cortical development.

## Discussion

AS is highly pervasive in the developing brain, generating proteomic diversity that is indispensable for normal neurodevelopment. Consistently, disruptions of splicing regulation have been implicated in a broad spectrum of NDDs.^24^ Although the existence of neuron-specific RNA isoforms is well established, how AS is regulated and the functional impact of many neural splice variants on neuronal differentiation, polarity, and morphology remain poorly understood. We previously identified biallelic *RBM42* pathogenic variants as the cause of a syndromic recessive NDD characterized by severe cortical malformations, growth impairment, and congenital defects.^25^ In this study, our findings support RBM42 as an important neurodevelopmental regulator that associates with hnRNP K and EFTUD2 and contributes to the cross-species regulation of AS-associated gene networks via recognition of conserved binding motifs. RBM42 loss is associated with disrupted splicing of cytoskeletal genes essential for neuronal architecture, together with cortical malformations in mice that resemble those observed in an individual carrying biallelic *RBM42* pathogenic variants.

By integrating genome-wide approaches, the splicing targets, RNA-binding sites, and candidate RBM42-associated proteins during neurodevelopment were defined. Hundreds of genes underwent significant AS changes in each experimental system, with 87 human-mouse ortholog pairs showing significant AS changes in both datasets (Figure 5D and Table S6). This broad effect aligns with accumulating evidence that dysregulated AS and RBP dysfunction are common pathological features of NDDs.^9^^,^^24^^,^^38^^,^^39^ Notably, the RBM42-dependent splicing program was strongly enriched for cytoskeletal genes (Figure 5E), implicating splicing dysregulation as a candidate contributor to the cytoskeletal abnormalities observed in RBM42-deficient neural cells and brain tissues. This aligns with previous studies demonstrating that RBP-regulated splicing programs govern key developmental processes such as cortical lamination. Together, these findings nominate cytoskeletal gene mis-splicing as an important candidate mechanism contributing to RBM42-related neurodevelopmental disease.

RBM42 is a core spliceosomal component, residing in the U4/U6·U5 tri-snRNP and pre-B complex, where it stabilizes RNA structures essential for spliceosome assembly.^26^^,^^27^^,^^40^ This intrinsic role points to functions in global splicing regulation that extend beyond the RNAs detected by RIP-seq. Consistently, only ∼21% of genes with RBM42-dependent alternative splicing in SH-SY5Y cells (113 of 526) overlapped with genes whose transcripts contained reproducible RBM42 RIP-seq peaks (Figure 5A and Table S4), suggesting that RBM42-dependent splicing changes may involve both RNA-associated and downstream mechanisms, although the overlap alone does not distinguish direct from indirect regulation. Notably, these candidate targets were strongly enriched for cytoskeletal pathways (Figure 5B), supporting a model in which RBM42 confers selective regulation over transcripts critical for neuronal architecture and morphogenesis. Dysregulation of these events is therefore likely to contribute to the widespread cellular dysfunction observed in RBM42 deficiency.

Among the candidate RBM42-associated splicing targets, *NIN* and *NUMA1* emerged as microtubule-related candidates in neural development. *NIN* encodes a centrosomal protein that anchors microtubules and promotes axon extension and branching downstream of SIP1.^41^ In C. elegans, the NINEIN homolog NOCA-2 acts in parallel with the CAMSAP homolog PTRN-1 to establish dendritic microtubule polarity.^42^ *NIN* exon 18 and exon 29 are developmentally regulated in opposite cell-type patterns; exon 18 is progenitor-enriched, whereas exon 29 is neuron-enriched.^21^ Alternative splicing of *NIN*, particularly at exons 18 and 29, may alter domains required for centrosomal anchoring, leading to disorganized microtubule minus-end arrays and axon polarity defects. *NUMA1* encodes the nuclear mitotic apparatus protein, essential for spindle organization and post-mitotic processes including axon growth and microtubule assembly; its dysregulation has been linked to neurodevelopmental defects in Huntington’s disease models.^36^ Aberrant *NUMA1* splicing could disrupt its interaction with dynein-microtubule complexes, resulting in impaired spindle orientation and aberrant axonal microtubules. Notably, disruption of cytoskeletal regulators and microtubule dynamics is a recurrent cause of NDDs.^43^^,^^44^^,^^45^ Consistent with this paradigm, our findings identify NIN and NUMA1 as RBM42-regulated cytoskeletal genes with AS changes in both species.

The ASO rescue experiments provide functional evidence that RBM42-dependent alternative splicing contributes to cytoskeletal organization and proliferative capacity. Although both *NIN*- and *NUMA1*-targeting ASOs corrected their respective splicing defects, only *NIN* exon 18 correction partially normalized the βIII-tubulin phenotype and partially restored the Ki67-positive fraction. Consistently, NINΔE18, but not NIN+E18, ameliorated the cytoskeletal abnormality. These findings identify *NIN* exon 18 mis-splicing as a downstream effector of RBM42 deficiency. The incomplete rescue and persistent TUNEL positivity indicate that *NIN* represents one component of a broader RBM42-regulated splicing network rather than the sole mediator of the cellular phenotype. Thus, the rescue data strengthen the central model that RBM42 couples alternative splicing to cytoskeletal organization during cortical development, while additional targets likely contribute to cell survival and the broader cortical abnormalities.

Our exploratory IP-MS analysis nominated multiple spliceosomal factors as candidate RBM42-associated proteins, including SF3B components and EFTUD2 (Figures S7D and S7G). EFTUD2 is a core component of the U5 snRNP^46^ and contributes to spliceosome remodeling and pre-mRNA splicing.^47^ Notably, pathogenic variants in *EFTUD2* cause mandibulofacial dysostosis with microcephaly, a developmental syndrome characterized by microcephaly, craniofacial anomalies, and developmental delay and intellectual disability of variable severity.^48^^,^^49^^,^^50^ Purkinje-cell-specific Eftud2 deficiency causes ferroptotic degeneration, cerebellar atrophy, and motor dysfunction in mice.^51^^,^^52^ The convergence of RBM42 and EFTUD2 in neurodevelopmental phenotypes suggests potential overlap in their splicing targets. RBM42 binding sites on pre-mRNAs may coincide with those of EFTUD2 or hnRNP K, indicating possible cooperative or antagonistic regulation. Future work should investigate such crosstalk to reveal additional layers of splicing control in brain development.

In conclusion, our findings establish RBM42 as a key regulator of the post-transcriptional network underlying cortical development. By coupling AS programs to cytoskeletal organization, RBM42 provides a mechanistic link between RNA processing and the structural assembly of the developing brain. This work addresses a long-standing gap in understanding how neuron-specific splicing regulation shapes neurodevelopmental architecture and disease, while also laying the foundation for targeted strategies to restore splicing fidelity in RBM42-related and other splicing-associated NDDs.

### Limitations of the study

The use of SH-SY5Y neuroblastoma cells may not fully capture the physiology of normal neural progenitors, potentially affecting the relevance of some findings. In addition, although apoptotic cells were observed in both PAX6-positive progenitor/radial glial populations and βIII-tubulin-positive cortical regions, the cell-type-specific functions of RBM42 during cortical development remain to be further elucidated using more lineage-restricted Cre drivers, such as NeuroD1-Cre or hGFAP-Cre. Mechanistically, we have not fully defined all *cis*-regulatory elements RBM42 recognizes. While we identified hundreds of RBM42-dependent splicing events, we primarily validated key candidates such as *NIN* and *NUMA1*. Although the ASO rescue experiments corrected specific splicing events in SH-SY5Y cells, they did not identify the full spectrum of RBM42-dependent splicing events underlying the TUNEL phenotype or demonstrate *in vivo* rescue of cortical abnormalities. Thus, other functionally important RBM42-regulated targets likely remain to be identified. Furthermore, although we identified interactions with partners such as hnRNP K and EFTUD2, the exact mechanisms and functional consequences of these complexes in directing splicing during neurodevelopment remain incompletely defined. Determining whether these interactions are cooperative or antagonistic represents an essential direction for future research to fully elucidate the RBM42-regulated splicing network. Because the sex of embryos and neonatal mice was not recorded, the potential influence of sex on the results of the mouse experiments could not be assessed.

## Resource availability

### Lead contact

Further information and requests for resources and reagents should be directed to and will be fulfilled by the lead contact, Jinglan Zhang (jinglanzhang@foxmail.com or jinglanzhang@zju.edu.cn).

### Materials availability

All unique/stable reagents in this study can be generated using protocols detailed in the Methods section or can be requested from the lead contact with a completed materials transfer agreement.

### Data and code availability

• The mass spectrometry proteomics data are publicly available through iProX and ProteomeXchange (iProX: IPX0014407000; ProteomeXchange: PXD071249).
• The SH-SY5Y bulk RNA-seq, mouse brain bulk RNA-seq, SH-SY5Y RBM42 RIP-seq, and E18.5 mouse cortical RBM42 RIP-seq datasets are publicly available in the Gene Expression Omnibus (GEO: GSE311526, GSE311557, GSE311642, and GSE337564), respectively.
• This paper does not report original code.
• Any additional information required to reanalyze the data reported in this paper is available from the lead contact upon request.

## Acknowledgments

The study was supported by the 10.13039/501100012166National Key R&D Program of China (2023YFC2705601, 2023YFC2705600, 2020YFA0804001, 2020YFA0804000) and by the 10.13039/501100001809National Natural Science Foundation of China (82571908, 82271898).

## Author contributions

The study was designed by B.X.Y., J.L.Z., and H.F.H. B.X.Y., Q.X.X., W.L.L., Y.L.C., and R.Q.S. performed experiments. B.X.Y., X.Q., and Y.Y.C. conducted bioinformatic analyses, with supervision and input from J.L.Z. Y.W.J., Y.L.W., J.W., H.X., and J.L.Z. contributed to the review and editing of the paper.

## Declaration of interests

The authors declare no competing interests.

## Declaration of generative AI and AI-assisted technologies in the manuscript preparation process

During preparation of the final-format manuscript, the authors used OpenAI Codex to assist with reformatting, language editing, and organization according to the iScience STAR Methods template. The author(s) reviewed and edited the output as needed and take full responsibility for the content of the published article.

## STAR★Methods

### Key resources table

**Table:** 

REAGENT or RESOURCE	SOURCE	IDENTIFIER
**Antibodies**		

Anti-RBM42	Abcam	Cat#ab241504
Immunoprecipitation-grade anti-RBM42	Bethyl Laboratories	Cat#A305-139A; RRID: AB_2631534
Anti-NIN	Proteintech	Cat#13007-1-AP; RRID: AB_3085405
Anti-NUMA1	Abcam	Cat#ab109262; RRID: AB_10863599
Anti-α-tubulin	Proteintech	Cat#80762-1-RR; RRID: AB_2918911
Anti-rabbit IgG, HRP-linked Antibody	Cell Signaling Technology	Cat#7074; RRID: AB_2099233
Anti-mouse IgG, HRP-linked Antibody	Cell Signaling Technology	Cat#7076; RRID: AB_330924
Anti-Rabbit Secondary HRP Antibody (for Wes)	ProteinSimple	Cat#042–206; RRID: AB_2860577
Anti-hnRNP K	Santa Cruz	Cat#sc-28380; RRID: AB_627734
Anti-EFTUD2	Proteintech	Cat#10208-1-AP; RRID: AB_2095834
Anti-β-tubulin	Proteintech	Cat#80713-1-RR; RRID: AB_2918906
Anti-βIII-tubulin	Servicebio	Cat#GB12139-100
Anti-Ki67	Servicebio	Cat#GB111499-100; RRID:AB_2927572
Donkey anti-Rabbit IgG (H + L) Highly Cross-Adsorbed Secondary Antibody, Alexa Fluor™ 555	Invitrogen	Cat#A-31572; RRID: AB_162543
Donkey anti-Mouse IgG (H + L) Highly Cross-Adsorbed Secondary Antibody, Alexa Fluor™ 488	Invitrogen	Cat#A-21202; RRID: AB_141607
Anti-CD31	Servicebio	Cat#GB11063-2-100; RRID: AB_2922436
Anti-ZO-1	Servicebio	Cat#GB115686-100; RRID: AB_3751487
Anti-SATB2	Servicebio	Cat#GB111449-100; RRID: AB_3719475
Anti-CTIP2	Abcam	Cat#ab18465; RRID: AB_2064130
Anti-TBR1	Servicebio	Cat#GB111317-100
Anti-phospho-histone H3	Cell Signaling Technology	Cat#9701; RRID: AB_331535
Anti-PAX6	Servicebio	Cat#GB150192-100
Anti-Vimentin	Servicebio	Cat#GB11192-100; RRID: AB_2814685
Anti-MAP2	Proteintech	Cat#17490-1-AP; RRID: AB_2137880
Anti-FLAG	Proteintech	Cat# 66008-4-Ig; RRID: AB_2918475
Anti-Histone H3	Cell Signaling Technology	Cat#4499; RRID: AB_10544537
Anti-β-Actin	Proteintech	Cat#66009-1-Ig; RRID: AB_2687938
Rabbit IgG (Supplied with the EZ-Magna RIP kit)	Millipore	Cat#17–701; component Part#PP64B; RRID: AB_97852

**Bacterial and virus strains**		

Trans5α Chemically Competent Cell	TransGen Biotech	CD201-01

**Biological samples**		

E12.5, E14.5, and E18.5 mouse brain/cortical tissue	This paper	N/A
P0.5 mouse brain/cortical tissue	This paper	N/A

**Chemicals, peptides, and recombinant proteins**		

DMEM/Nutrient Mixture F-12	Gibco	Cat#11320033
Fetal bovine serum	Gibco	Cat#A5256701
Puromycin	Sangon	Cat#E607054
RNAiso Plus	Takara	Cat#9109
Protein A/G PLUS-Agarose	Santa Cruz	Cat#sc-2003
Phalloidin	Servicebio	Cat#G1249
DAPI	Beyotime	Cat#C1006
4% paraformaldehyde	Servicebio	Cat#G1101
Penicillin–streptomycin	Gibco	Cat#15140122
Lipofectamine 3000	Invitrogen	Cat#L3000150
RIPA	Beyotime	Cat#P0013B
Nissl Staining Kit	Beyotime	Cat#C0117
EdU Imaging Kit	Yeasen	Cat#40275ES60

**Critical commercial assays**		

PrimeScript RT reagent Kit with gDNA Eraser	Takara	Cat#RR047A
TB Green Premix Ex Taq	Takara	Cat#RR420A
BCA Protein Assay Kit	Takara	Cat#T9300A
Nuclear and Cytoplasmic Protein Extraction Kit	Beyotime	Cat#P0027
xGen Broad-Range RNA Library Prep Kit	Integrated DNA Technologies	Cat#10009865
Magna RIP RNA-Binding Protein Immunoprecipitation Kit	Millipore	Cat#17–701
TSAPLus Fluorescent Kit	Servicebio	Cat#G1236
TUNEL assay kit	Epizyme	Cat#CX108
SimpleWestern 66–440 kDa separation plate	Bio-Techne	Cat#SM-W005

**Deposited data**		

Mass spectrometry proteomics data	ProteomeXchange/iProX	ProteomeXchange: PXD071249; iProX: IPX0014407000
SH-SY5Y bulk RNA-seq	Gene Expression Omnibus	GEO: GSE311526
Mouse brain bulk RNA-seq	Gene Expression Omnibus	GEO: GSE311557
RBM42 RIP-seq in SH-SY5Y cells	Gene Expression Omnibus	GEO: GSE311642
RBM42 RIP-seq in E18.5 mouse cortex	Gene Expression Omnibus	GEO: GSE337564

**Experimental models: Cell lines**		

SH-SY5Y neuroblastoma cells	National Collection of Authenticated Cell Cultures, Chinese Academy of Sciences	Cat#SCSP-5014
293T cells	National Collection of Authenticated Cell Cultures, Chinese Academy of Sciences	Cat#SCSP-502

**Experimental models: Organisms/strains**		

Mouse: *Rbm42*^flox/flox^: C57BL/6J background	Southern Model Organisms	N/A
Mouse: Rbm42 conditional knockout: *Rbm42*^flox/flox^; Nestin-Cre^+^	This study	N/A

**Oligonucleotides**		

Primers, gRNAs, shRNAs, and splice-switching ASOs	This paper	See Table S1

**Recombinant DNA**		

Construct GV394: CMV-MCS-SV40-Neomycin	GeneChem	https://www.genechem.com.cn/
Construct GV394: CMV-MCS-RBM42-SV40-Neomycin	GeneChem	https://www.genechem.com.cn/
Construct KV562: CMV-MCS-polyA-EF1A-zsGreen-sv40-Neomycin	GeneChem	https://www.genechem.com.cn/
Construct KV562: 3xFLAG-NIN delta E18 expression	GeneChem	https://www.genechem.com.cn/
Construct KV562: 3xFLAG-NIN plus E18 expression	GeneChem	https://www.genechem.com.cn/
Construct GV112: hU6-shNC-CMV-Puromycin	GeneChem	See Table S1
Construct GV112: hU6-shRBM42-CMV-Puromycin	GeneChem	See Table S1

**Software and algorithms**		

featureCounts v1.5.0-p3	Subread project	https://subread.sourceforge.net/
SAMtools	SAMtools project	https://www.htslib.org/
Cutadapt v1.18 (RIP-seq)	Marcel Martin	https://cutadapt.readthedocs.io/
HISAT2 v2.0.5 (bulk RNA-seq)	HISAT2 project	https://daehwankimlab.github.io/hisat2/
HISAT2 v2.2.1 (RIP-seq)	HISAT2 project	https://daehwankimlab.github.io/hisat2/
fastp v0.19.7	OpenGene	https://github.com/OpenGene/fastp
FastQC v0.11.5	Babraham Bioinformatics	https://www.bioinformatics.babraham.ac.uk/
FastQ Screen v0.13.0	Babraham Bioinformatics	https://www.bioinformatics.babraham.ac.uk/
ImageJ v1.54g	National Institutes of Health	https://imagej.nih.gov/ij/
NeuronJ	ImageScience	https://imagescience.org/
GraphPad Prism v10.1.2	GraphPad Software	https://www.graphpad.com/
rMATS v4.1.0 (SH-SY5Y RNA-seq)	Shen et al.^53^	https://rnaseq-mats.sourceforge.net/
rMATS v4.2.0 (mouse brain RNA-seq)	Shen et al.^53^	https://rnaseq-mats.sourceforge.net/
R v4.6.0	R Foundation for Statistical Computing	https://www.r-project.org/
clusterProfiler v4.20.0	Bioconductor	https://bioconductor.org/packages/clusterProfiler/
rrvgo v1.24.0	Bioconductor	https://bioconductor.org/packages/rrvgo/
MaxEntScan	Yeo and Burge^34^	http://hollywood.mit.edu/burgelab/maxent/
MACS v1.4.2	MACS project	https://github.com/macs3-project/MACS
MEME Suite v5.3.0 (DREME)	MEME Suite	https://meme-suite.org/meme/tools/dreme
Compass for Simple Western v7.0.0	Bio-Techne	N/A
NCBI Conserved Domain Database	National Center for Biotechnology Information	https://www.ncbi.nlm.nih.gov/Structure/cdd/
Proteome Discoverer v2.4	Thermo Fisher	https://www.thermofisher.cn/

**Other**		

Wes Simple Western instrument	ProteinSimple; Bio-Techne	N/A
BTX Gemini electroporation system	BTX	N/A
SC3000 label-free live-cell microscopy system	Zircon Optoelectronics (Suzhou) Co., Ltd.	Model: SC3000

### Experimental model and study participant details

#### Ethics statement

All animal procedures were performed in accordance with the ethical guidelines and regulations of the Institute of Shanghai Model Organisms Center, Inc. The study was approved by the Ethics Review Committee at the International Peace Maternity and Child Health Hospital affiliated with Shanghai Jiao Tong University School of Medicine (GKLW 2020–42). The study did not enroll human participants or use identifiable primary human material.

#### Mouse models

*Rbm42*^flox/+^ mice were bred on a C57BL/6J background and generated using the CRISPR/Cas9 system. All animal procedures were performed following the ethical guidelines and regulations of Institute of Shanghai Model Organisms Center, Inc. Mice were maintained under specific pathogen-free conditions at 22°C–25°C and 50–60% relative humidity on a 12 h light-dark cycle, with food and water available *ad libitum*. Timed matings were established by overnight co-housing of male and female mice, and noon of the day on which a vaginal plug was detected was designated E0.5. The sex of embryos and neonatal mice was not recorded, and analyses were not stratified by sex. Animals were euthanized by cervical dislocation before tissue collection. The mouse *Rbm42* gene is encoded by 10 exons in the genomic locus. We constructed a targeting vector containing *loxP* sites (triangles) flanking exons 3 and 4 of the *Rbm42* gene (Figure S1A). Two guide RNAs (gRNAs) were designed to target genomic sites flanking exons 3–4 of the *Rbm42* gene to enable *loxP* insertion. The gRNA oligo sequences were as follows: gRNA1: GAGTCAGAAAAGTTGATCCTGGG; gRNA2: CTAGACAGCCCGTCTCCCCTCGG. These gRNAs and Cas9 mRNA were electroporated into C57BL/6J mouse embryonic stem (ES) cells. Resulting chimeric founder mice were generated and subsequently crossed with C57BL/6J mice to produce heterozygous *Rbm42*^flox/+^ mice. In this way, we developed a specific mouse strain that harbored conditional alleles of the *Rbm42* gene. Homozygous *Rbm42*^flox/flox^ mice were obtained by intercrossing heterozygous *Rbm42*^flox/+^ mice. After establishment and validation of the floxed line, Nestin-Cre mice, in which Cre recombinase expression is driven by the Nestin promoter,^54^ were used for conditional deletion of *Rbm42*. To generate experimental littermates, *Rbm42*^flox/flox^ mice were crossed with *Rbm42*^flox/+^; Nestin-Cre^+^ mice. In this study, control littermates (*Rbm42*^flox/flox^, Nestin-Cre^-^) were used as controls and are referred to as Ctrl throughout the text and Figures. Littermates carrying the *Rbm42*^flox/flox^; Nestin-Cre^+^ genotype were used as the Rbm42 conditional knockout group and are referred to as cKO throughout the text and figures. Genotyping was conducted according to standard protocols, and the primers used are listed in Table S1A. Embryos were analyzed at E12.5, E14.5, and E18.5 and neonatal mice at P0.5, as specified in the Results and figure legends.

#### Cell lines

Human embryonic kidney 293T (HEK-293T) cells and human SH-SY5Y neuroblastoma cells, both of female donor origin, were obtained from the National Collection of Authenticated Cell Cultures, Chinese Academy of Sciences (Shanghai, China). HEK-293T cells were maintained in DMEM containing 10% fetal bovine serum and 1% penicillin-streptomycin. SH-SY5Y cells were propagated in Dulbecco’s modified Eagle’s medium (DMEM)/Nutrient Mixture F-12 supplemented with 10% fetal bovine serum (FBS). All cells were incubated at 37°C in a humidified atmosphere with 5% CO2. The cell lines were authenticated by short tandem repeat (STR) analysis and tested negative for mycoplasma contamination.

### Method details

#### Lentiviral shRNA delivery and stable cell line generation

Target-specific lentiviral shRNA particles were designed and synthesized by Genechem (Shanghai, China). The shRNA sequence targeting RBM42 was designated shRBM42. The control shRNA group was designated shNC. Recombinant lentiviral particles were produced in HEK-293T cells. Viral supernatants were harvested at 48 and 72 h post-transfection, concentrated by ultracentrifugation, and titered via qPCR (3.5 × 10^8^ TU/mL). For transduction, cells were seeded at 50% confluence in 6-well plates. Viral particles were added at a multiplicity of infection (MOI) of 2. After 12 h, the viral medium was replaced with fresh complete medium. Transduced cells were selected using 3 μg/mL puromycin (Sangon) for 7 days. Fresh puromycin-containing medium was replenished every 48 h. Surviving cells were expanded and maintained in 1 μg/mL puromycin for subsequent experiments. Knockdown efficiency was validated via quantitative RT-PCR (for mRNA) and western blotting (for protein). The shRNA sequences are listed in Table S1B.

#### Southern blot

Genomic DNA isolated from mice was analyzed by Southern blotting according to the standard procedure of the Institute of Shanghai Model Organisms Center, Inc. For validation of the 5′ and 3′ regions of the targeted *Rbm42* allele, genomic DNA was digested overnight at 37°C with PsiI or NdeI, respectively. The digested DNA was separated by agarose gel electrophoresis and transferred to a positively charged nylon membrane by capillary transfer in 20× SSC. Probe-1 and Probe-2 were generated by PCR and labeled by incorporation of DIG-dUTP. Membranes were hybridized with the corresponding DIG-labeled probes at 50°C overnight. After hybridization, the membranes were washed twice with 2× SSC containing 0.1% SDS at room temperature, followed by two high-stringency washes with 0.5× SSC containing 0.1% SDS at 65°C. Hybridization signals were detected using an alkaline phosphatase-conjugated anti-DIG antibody and CDP-Star chemiluminescent substrate and recorded on X-ray film. For the PsiI/Probe-1 assay, the expected fragment sizes were 6.3 kb for the wild-type allele and 4.6 kb for the targeted allele. For the NdeI/Probe-2 assay, the predicted wild-type and targeted fragments were 9.0 and 9.1 kb, respectively; these fragments were not resolved under the electrophoresis conditions used and appeared as an approximately 9.0–9.1-kb signal.

#### RNA isolation, reverse transcription PCR, and quantitative real-time PCR (qPCR)

Total RNA was extracted from cells and tissues using RNAiso Plus (Takara) according to the manufacturer’s instructions. A total of 1 μg RNA was used for cDNA synthesis using the PrimeScript RT reagent Kit with gDNA Eraser (Takara). The cDNA products were subjected to RT-PCR analysis and the products were separated on a 2% agarose gel. Primers are listed in Tables S1C and S1D. mRNA levels were measured by qPCR using TB Green Premix Ex Taq (Takara) on ABI 7500 (Applied Biosystems). *ACTB* was used as the reference gene, and relative expression was calculated using the 2^−ΔΔCt^ method. Primers are listed in Table S1E.

#### Western blotting

For protein extraction, cells were lysed in 1× RIPA (50 mM Tris pH 7.4, 150 mM NaCl, 1% Triton X-100, 1% sodium deoxycholate and 0.1% SDS) with EDTA-free Protease Inhibitor Cocktail (Roche). The cells were placed on ice and allowed to lyse for at least 10 min, sonicated if needed and centrifuged at 12,000 × g for 15 min. The concentration of the protein was determined utilizing the BCA Protein Assay Kit (Takara). Cell lysates were subjected to denaturation utilizing a 5 × SDS sample buffer and subsequently examined through a standard western blot employing targeted antibodies (RBM42 antibody, ab241504). The images were evaluated using ImageJ.

#### Quantitative size-based capillary western (WES) blot assays

Protein concentration was quantified by BCA (Takara) and 2 μg were loaded on a SimpleWestern 66–440 kDa separation plate (Bio-Techne) according to the protocol of ProteinSimple WES system.^55^ For detection of Nin and Numa1, following antibodies were used: NIN (1:50, Proteintech) and NUMA1 (1:50, Abcam). The 66–440-kDa separation module was also used to detect α-tubulin as a loading control. Although α-tubulin lies below the nominal separation range, its signal was detected under the assay conditions used. Anti-Rabbit Secondary HRP Antibody (042–206; ProteinSimple; Bio-Techne; provided as working stock dilution from manufacturer) was used as a secondary antibody. The samples were acquired on a Wes instrument (ProteinSimple; Bio-Techne). Signals were visualized as blot images, and the intensity of the signal was used to quantify relative abundance of protein using the Compass for SW software (ProteinSimple version 7.0.0).

#### Co-immunoprecipitation

SH-SY5Y cells were rinsed with ice-cold PBS and subsequently collected in 500 μL of NETN lysis buffer (250 mmol/L NaCl, 5 mmol/L EDTA, 50 mmol/L Tris-HCl, 0.5% NP-40) supplemented with protease inhibitor tablets (Roche). Following 15 min of incubation in lysis buffer at 4°C, the resulting lysate was subjected to centrifugation for 5 min at 16,000 × g at 4°C. A total of 1 mg of protein was utilized for the immunoprecipitation process at 4°C. Subsequently, 2 μg of the RBM42 antibody was introduced to the supernatant and allowed to incubate overnight at 4°C. Then, 20 μL of Protein A/G Plus Agarose (Santa Cruz) was added, and the mixture was incubated for 1 h at 4°C. Samples were then centrifuged 5 min at 1000 × g at 4°C and washed five times with 1 mL of lysis buffer. Immunoprecipitated proteins were eluted by addition of 30 μL of 1× SDS sample buffer and subsequently incubating at 100°C for 10 min. The initial lysates, along with the proteins that were immunoprecipitated, underwent analysis through immunoblotting utilizing targeted antibodies. The following antibodies were used: anti-RBM42, anti-hnRNP K, anti-EFTUD2.

#### LC-MS/MS analysis

Nuclear proteins of SH-SY5Y cells were extracted using Nuclear and Cytoplasmic Protein Extraction Kit (Beyotime). RBM42 antibody (Bethyl) was added to lysates with Protein A/G agarose and incubated at room temperature for 1 h. The Protein A/G beads that were incubated with the IgG antibody served as the negative control. The bead-bound proteins were washed with lysis buffer for four times. Samples were eluted using elution buffer and subsequently underwent Western blotting or in-gel digestion. LC-MS/MS was conducted by Jingjie PTM BioLabs. RBM42 and matched IgG immunoprecipitates were separated by SDS-PAGE and subjected to in-gel tryptic digestion following reduction with dithiothreitol and alkylation with iodoacetamide. Desalted peptides were analyzed using an EASY-nLC 1000 system coupled to a Q Exactive Plus mass spectrometer in data-dependent acquisition mode, with the 20 most abundant precursor ions selected for HCD fragmentation. MS/MS data were searched using Proteome Discoverer. Searches allowed Trypsin/P specificity, up to two missed cleavages, precursor and fragment mass tolerances of 10 ppm and 0.02 Da, carbamidomethylation of cysteine as a fixed modification, and methionine oxidation and protein N-terminal acetylation as variable modifications. High-confidence peptide identifications with ion scores >20 were retained. Proteins detected in the IgG control were excluded, and the remaining proteins were reported as candidate RBM42-associated proteins. The 81-protein IP-only set, including the bait protein RBM42, was used for enrichment and network analyses. Selected candidates were further assessed by co-immunoprecipitation followed by immunoblotting where indicated. IP–MS was performed in one biological experiment, with RBM42 and IgG immunoprecipitations processed in parallel.

#### RNA-seq and data analysis

Two biological replicates of RNA samples per condition were obtained from SH-SY5Y cells expressing shRBM42 or shNC. Three biological replicates per group were obtained from the brains of Rbm42 cKO and control mice. More than 1 μg of total RNA per sample was used for RNA-seq. Poly(A)+ RNA was enriched using oligo(dT)-coupled magnetic beads and fragmented for cDNA synthesis. RNA-seq libraries were prepared using the xGen Broad-Range RNA Library Prep Kit (IDT) and sequenced on an Illumina NovaSeq 6000 platform to generate 150-bp paired-end reads at Novogene. For both datasets, raw reads were processed using fastp v0.19.7. Only the resulting clean reads were used for downstream analyses. Clean reads from SH-SY5Y cells were aligned to the human GRCh38 reference genome, whereas reads from mouse brain samples were aligned to the mouse GRCm39 reference genome. Alignment was performed using HISAT2 with default parameters. The resulting SAM files were converted to BAM format using SAMtools, and the BAM files were used as input for alternative splicing analysis. Sequencing quality was assessed based on the sequencing error rate, Q20 and Q30 percentages, GC content, and genome-mapping statistics. For analyses requiring gene-level expression estimates, read counts were obtained using featureCounts v1.5.0-p3. Reads with mapping quality scores below 10, improperly paired reads, and reads mapped to multiple genomic locations were excluded. FPKM values were calculated by normalizing read counts for sequencing depth and gene length.

#### Alternative splicing analysis

Alternative splicing (AS) events were systematically identified and quantified using rMATS (Replicate Multivariate Analysis of Transcript Splicing).^53^ rMATS categorized AS events into five canonical types: skipped exon (SE), alternative 5′ splice site (A5SS), alternative 3′ splice site (A3SS), mutually exclusive exons (MXE), and retained intron (RI). For each AS event and biological replicate, rMATS calculated the inclusion level (Percent Spliced In, PSI) from effective-length-normalized inclusion and skipping counts as follows: PSI = (IJC/IncFormLen)/[(IJC/IncFormLen) + (SJC/SkipFormLen)], where IJC and SJC denote the inclusion and skipping counts, respectively, and IncFormLen and SkipFormLen denote the corresponding effective lengths used for normalization. IncLevel1 and IncLevel2 in the rMATS output contain the replicate-specific inclusion levels for group 1 (the experimental group: shRBM42 or *Rbm42* cKO) and group 2 (the control group: shNC or control), respectively. The group-level difference in inclusion was reported by rMATS as IncLevelDifference and calculated as follows: IncLevelDifference = mean(IncLevel1) − mean(IncLevel2). Thus, positive IncLevelDifference values indicate higher inclusion in the experimental group, whereas negative values indicate lower inclusion in the experimental group relative to the corresponding control group. Biological replicate information (*n* = 2 per group in SH-SY5Y cells and *n* = 3 per group in mouse brains) was incorporated into the rMATS statistical model for estimating *p* values, which were adjusted for multiple testing using the Benjamini–Hochberg procedure to obtain FDR values. Events with FDR <0.05 and |IncLevelDifference| > 0.1 were considered statistically significant. Significant AS events were extracted and imported into R for downstream analysis. Functional annotation of affected genes was performed using clusterProfiler against the GO and KEGG databases. Enriched GO biological process terms associated with the conserved human–mouse AS gene set were further summarized using rrvgo. Terms with a Benjamini–Hochberg-adjusted *p* value <0.05 were grouped according to semantic similarity using the Rel method and a similarity threshold of 0.7. Neurodevelopment-related terms were identified by keyword-based annotation, and the ten most significant terms were labeled in the semantic scatterplot.

#### Cross-species identification of conserved AS genes

Significant AS genes were defined separately in humans and mice as genes with at least one AS event meeting FDR <0.05 and |IncLevelDifference| > 0.1, with multiple events collapsed by Ensembl Gene ID. Human–mouse orthology was determined using Ensembl Compara release 116, and gene symbols were standardized according to current Ensembl, HGNC, and MGI nomenclature. The conserved AS gene set comprised genes showing significant AS in both species and linked by a high-confidence one-to-one orthology relationship. One-to-many, many-to-many, and low-confidence mappings were excluded without manual resolution, and duplicate mappings were collapsed by paired human and mouse Ensembl Gene IDs.

#### Densitometric analysis of RT-PCR products

Gel images were analyzed using ImageJ. Band intensities corresponding to exon-included and exon-skipped products were measured after background subtraction using identical region of interest settings for each lane. A semiquantitative exon-inclusion index was calculated as inclusion-band intensity/(inclusion-band intensity + skipping-band intensity). Because endpoint RT-PCR band intensity can be affected by amplicon length and amplification efficiency—particularly for NIN, whose products differ substantially in size—this index was used only for within-assay relative comparisons and was not interpreted as an absolute PSI value. *HPRT*1 was used as an internal control for endpoint RT-PCR. Quantification was performed on independent biological replicates (*n* = 2 or 3) as indicated in the figure legends.

#### Disease gene set enrichment analysis

Over-representation analysis was performed to assess whether conserved RBM42-regulated targets were enriched for neurodevelopmental DDG2P genes, defined as the intersection of DDG2P genes and green-rated genes in the Genomics England PanelApp Intellectual disability panel 285. Gene identifiers were mapped to human Entrez IDs, and the background universe comprised 11,275 Entrez-mapped genes detected in the SH-SY5Y RNA-seq dataset (FPKM ≥1), of which 1,068 were neurodevelopmental DDG2P genes. Of the 87 conserved targets, 79 were represented in this background and included in the enrichment test. The 2 × 2 table comprised 14 targets within and 65 targets outside the neurodevelopmental DDG2P gene set, and 1,054 genes within and 10,142 genes outside this set among the remaining background genes. Enrichment was evaluated using a two-sided Fisher’s exact test; the odds ratio and *p* value are reported.

#### RIP-seq data processing

To purify RNAs bound to RBM42 protein in SH-SY5Y cells and E18.5 mouse cortical tissues, the Magna RIP RNA-Binding Protein Immunoprecipitation Kit (Millipore) was used. Briefly, cells or tissues were lysed in 200 μL RIP lysis buffer supplemented with RNase inhibitor and protease inhibitor. After 5 min of incubation on ice, 50 μL of magnetic bead suspension was added to each tube. The beads underwent two washing cycles utilizing 500 μL of RIP wash buffer. Subsequently, 5 μg of immunoprecipitation-grade anti-RBM42 (Bethyl) antibody (IP sample antibody) or Rabbit anti-IgG (mock sample antibody) was introduced to the beads and allowed to incubate at room temperature for 30 min. The beads were washed five times with wash buffer, and then resuspended in 900 μL of RIP immunoprecipitation buffer. For each RIP reaction, 900 μL of RIP immunoprecipitation buffer was freshly prepared by combining 860 μL RIP Wash Buffer, 35 μL 0.5 M EDTA, and 5 μL RNase inhibitor. The cleared RIP lysate was introduced to the antibody-bead complex and allowed to incubate overnight at 4°C. RNA purification was conducted utilizing a proteinase K buffer (RIP wash buffer, 10% SDS, and 10 mg/mL proteinase K). The RNA was precipitated using Salt Solution I, Salt Solution II, a Precipitate Enhancer, and absolute ethanol. The isolated RNA was used for either RIP-seq or qRT-PCR. IP, mock and input RNAs were shipped to Cloud-Seq Biotech Inc. (Shanghai, China) for library construction and sequencing or used for quantitative PCR analysis.

Raw paired-end reads were evaluated using Q30 statistics, FastQC, and FastQ Screen. Adapter sequences and low-quality reads were removed using Cutadapt. Three biological replicates were analyzed for each RIP-seq experiment. Clean reads from SH-SY5Y and mouse cortical RIP-seq libraries were aligned to hg38 and mm39/GRCm39, respectively, using HISAT2. RBM42-enriched regions were identified using MACS by comparing RBM42 immunoprecipitation libraries with the corresponding IgG controls. Peaks from the three biological replicates were compared at the genomic-coordinate level using replicate 1 as the reference. A peak was considered reproducible when it overlapped by at least 1 nucleotide with a peak in each of replicates 2 and 3. Overlapping peaks were not merged, and the replicate-1 coordinates were retained. Reproducible peaks were annotated against the corresponding hg38 or mm39/GRCm39 reference annotation, and peaks overlapping annotated exons of protein-coding transcripts were retained and assigned the corresponding gene and transcript identifiers. The reproducible peak set was used for transcript feature annotation, motif discovery, and downstream enrichment analysis. Motif analysis was performed using DREME on sequences extending 50 bp upstream and downstream of each peak summit.

#### RIP-qPCR analysis

For RIP-qPCR, SH-SY5Y cells or cerebral cortices dissected from E18.5 embryos were collected and RNAs extracted from the input samples and immunoprecipitates generated with anti-RBM42 (Bethyl) and anti-β-tubulin (Proteintech) or rabbit IgG antibodies were converted into cDNA and subjected to qPCR. Primers are listed in Table S1F. Three independent biological replicates were analyzed. Rabbit IgG served as a negative control, and β-tubulin immunoprecipitation was included as an additional negative IP control. For RIP-qPCR analyses shown in Figures 4M and 4N, RNA enrichment was expressed as percentage of input (% Input). For Figure 5C, enrichment was expressed as fold enrichment relative to the IgG negative control. Data from independent biological replicates were used for statistical analysis.

#### Immunofluorescence

Multiplexed immunofluorescence staining using tyramide signal amplification was performed with the TSAPlus Fluorescence Kit (Servicebio, China). SH-SY5Y cells were cultured on coverslips and fixed with 4% paraformaldehyde. Murine brain tissues were formalin-fixed, paraffin-embedded (FFPE), and sectioned at a thickness of 4 μm. Briefly, slides were dewaxed in xylene substitutes and rehydrated. Antigen retrieval was performed in citrate buffer using microwave heating (95°C, 20 min). Samples were incubated in 3% H_2_O_2_ for 25 min at room temperature to quench endogenous peroxidase activity. Sections were blocked with 3% BSA for 30 min. Primary antibodies were applied overnight at 4°C, followed by incubation with HRP-conjugated secondary antibodies for 50 min at room temperature. Tyramide-fluorophore conjugates (iF488, iF555, and iF647), diluted 1:500 in amplification buffer, were then applied. Between successive staining cycles, bound primary and secondary antibody complexes were gently removed using Immunostain Elution Buffer, while the covalently deposited tyramide-fluorophore signals were retained. The effectiveness of antibody stripping was verified in preliminary single-antibody control experiments. Nuclei were counterstained with DAPI. Images were acquired using a spectral confocal microscope with the following excitation/emission settings: DAPI, 359/457 nm; iF488, 491/516 nm; iF555, 557/570 nm; and iF647, 656/670 nm. For quantitative tissue imaging, anatomically matched coronal sections were analyzed using ImageJ with consistent ROI definitions across groups. SATB2-, CTIP2-, and TBR1-positive nuclei were counted within standardized cortical ROIs and expressed as cells per 200,000 μm^2^ at E18.5 or per 400-μm cortical width at E14.5. CD31-positive vascular profiles were manually counted within matched dorsal telencephalic or cortical ROIs and normalized to ROI area. PH3-positive nuclei along the ventricular surface were counted and expressed per 100 μm of ventricular surface. TUNEL-positive nuclei were counted within matched cortical ventricular/subventricular zone ROIs. Individual embryos were treated as independent biological replicates. For F-actin staining, fixed and permeabilized cells were incubated with fluorescently conjugated phalloidin. F-actin-positive area was quantified by fluorescence thresholding in ImageJ and normalized to the corresponding total cell area and then to the mean of the shNC group.

#### Nissl staining and morphometric analysis

Murine embryonic brain tissues were fixed, paraffin-embedded, and cut into 4 μm serial sections. After dewaxing and graded ethanol rehydration, sections were stained with Nissl solution for 5–10 min. Following brief rinsing and 95% ethanol differentiation, sections were dehydrated, cleared, and mounted for microscopic observation, which displayed typical blue-purple neuronal staining. For morphometric analysis, images were acquired via microscopy. Lateral ventricle area and cortical thickness were quantified on anatomically matched sections from individual embryos as biological replicates using ImageJ.

#### Label-free live cell microscopy system

This study employed the SC3000 label-free live cell microscopy system (Zircon Optoelectronics (Suzhou) Co., Ltd.) to observe samples, with its core imaging technology being intensity diffraction tomography (IDT). Three-dimensional label-free imaging of SH-SY5Y cells was performed using transport of intensity diffraction tomography with non-interferometric synthetic aperture (TIDT-NSA), as described by Li et al.^56^ When an incident beam illuminates transparent biological samples (e.g., live cells), localized refractive index (RI) differences in subcellular structures induce wavefront distortion in the transmitted light field. The system captures a series of two-dimensional intensity images via annular illumination scanning. By modeling the three-dimensional sample as a series of axially aligned two-dimensional slices and integrating phase transfer functions under varying annular illumination angles, a linear model is established between the sample’s phase information and the intensity images. This enables direct reconstruction of the sample’s three-dimensional refractive index distribution solely from the intensity images. Ultimately, this achieves quantitative, label-free, non-invasive, nanoscale characterization of optical properties (refractive index/thickness) in live cells. Neurite length was quantified from IDT images using ImageJ with the NeuronJ plugin. After spatial calibration using the image scale bar, clearly identifiable neurites were traced from the cell body to their visible endpoints. Trace lengths were converted to micrometers and averaged within each field.

#### Validation of neurite structures by MAP2 immunofluorescence

To confirm that the fine protrusions observed under IDT imaging represented bona fide neurite extensions, we performed MAP2 immunofluorescence staining on SH-SY5Y cells (Figure S6F). The co-localization between MAP2-positive neurites and the filamentous structures visualized by IDT confirmed that the refractive index–based protrusions captured in label-free images indeed correspond to neuronal processes.

#### Splice-switching ASO rescue assay

RBM42-deficient SH-SY5Y cells were transfected with either a control ASO or a splice-switching ASO targeting NIN exon 18 or NUMA1 exon 16 according to the manufacturer’s instructions (GenePharma). Cells were seeded on coverslips or culture plates one day before transfection to achieve 50%–70% confluency at transfection. ASOs were transfected at a final concentration of 100 nM using Lipofectamine 3000. Cells were harvested 48 h after transfection for RT-PCR and fixed at 48 h for βIII-tubulin immunofluorescence, 72 h for Ki67 immunofluorescence and TUNEL staining. All ASO rescue assays were performed in three independent biological experiments, with each experiment representing a separately cultured and transfected batch of cells. For imaging assays, field- and cell-level measurements were averaged within each experiment to generate one value per condition. These biological-replicate means, rather than individual fields or cells, were used as the statistical units for hypothesis testing. The control ASO was used as a non-targeting negative control. ASO sequences are listed in Table S1B.

#### Immunocytochemistry staining

Immunocytochemistry (ICC) staining was performed to detect cellular βIII-tubulin and Ki67 expression, and TUNEL staining was used to assess apoptosis. Cells cultured on coverslips were rinsed with ice-cooled PBS, fixed in 4% paraformaldehyde at room temperature, permeabilized with 0.5% Triton X-100, and blocked with 5% BSA to block non-specific binding. Subsequently, samples were incubated overnight at 4°C with primary antibodies: anti-βIII-tubulin (Servicebio, GB12139-100, 1:500) and anti-Ki67 (Servicebio, GB111499-100, 1:250). After PBS washing, fluorescent secondary antibodies (Invitrogen, A-31572, 1:500; Invitrogen, A-21202, 1:500) were applied at room temperature for 1 h in the dark. Nuclei were counterstained with DAPI, and coverslips were mounted with antifade medium for imaging. TUNEL staining was carried out using a commercial kit (Epizyme, CX108) following the manufacturer’s instructions, with cell fixation and permeabilization procedures consistent with ICC staining. Nuclei were counterstained with DAPI after the TUNEL reaction for subsequent microscopic analysis.

For quantitative analysis of cell-based assays, images from all groups within an independent experiment were acquired using identical microscope settings. Ki67 positivity was defined as a DAPI-positive nucleus with nuclear Ki67 fluorescence; TUNEL positivity was defined as a DAPI-positive nucleus showing intranuclear TUNEL fluorescence exceeding the corresponding background threshold. The Ki67-or TUNEL-positive fraction was calculated as the number of positive nuclei divided by the total number of DAPI-positive nuclei multiplied by 100. Three non-overlapping fields per condition were randomly selected across each coverslip using the DAPI channel; coverslip edges and fields containing obvious technical artifacts were excluded. For βIII-tubulin analysis, 5–20 non-overlapping fields per condition were randomly selected, single-cell regions of interest (ROIs) were manually delineated, and the thresholded βIII-tubulin-positive area was normalized to total cell area. Image analysis was performed using ImageJ.

#### EdU incorporation assay

SH-SY5Y cells were exposed to EdU at 10 μM for 2 h. EdU incorporation was detected using the YSFluor 488 EdU Imaging Kit (Yeasen) after fixation for 15 min and permeabilization with 0.5% Triton X-100 for 20 min, according to the manufacturer’s instructions. Nuclei were counterstained with DAPI. EdU-positive nuclei were quantified in 3 fields per biological replicate using identical imaging settings and independently cultured experiments were used as the statistical units.

#### NIN isoform-expression rescue assay

To assess the functional effect of distinct NIN splice isoforms, human NIN exon 18-skipped (NINΔE18) and exon 18-included (NIN+E18) expression constructs were generated by GeneChem (Shanghai, China). The NINΔE18 construct was based on human NIN transcript GenBank: NM_016350.5, encoding GenBank: NP_057434.4 (1,377 amino acids), whereas the NIN+E18 construct was based on GenBank: NM_182946.2, encoding GenBank: NP_891991.2 (2,090 amino acids). Pairwise sequence alignment confirmed that the two coding sequences differed by the 2,139-nucleotide exon 18-encoded segment, corresponding to 713 amino acids, and were otherwise identical. The corresponding NIN coding sequences were cloned into the KV562 mammalian expression vector using KpnI and PacI sites. The vector contains a CMV enhancer-driven multiple cloning site followed by polyA, and an independent EF1A-zsGreen-SV40-Neomycin cassette. Both NIN isoforms were expressed with an N-terminal 3×FLAG tag using the same vector backbone.

For isoform rescue experiments, RBM42-deficient SH-SY5Y stable cells were electroporated with empty KV562 vector, 3×FLAG-NINΔE18, or 3×FLAG-NIN+E18 plasmid. Briefly, cells were trypsinized, washed once with PBS, and resuspended at 4 × 10^6^ cells in 380 μL PBS. Five micrograms of plasmid DNA diluted in 20 μL TE buffer was added to the cell suspension to a final volume of 400 μL. The mixture was transferred into a disposable 2-mm electroporation cuvette and electroporated using a BTX Gemini electroporation system. Electroporation was performed using the built-in BTX Gemini SH-SY5Y preset exponential-decay program, with displayed settings of 150 V, 800 μF, R4 resistance, and a 2-mm electrode gap. After pulsing, cells were kept on ice for 5 min and then plated in complete culture medium for recovery.

For cytoskeletal rescue analysis, electroporated cells were fixed at 72 h after plasmid delivery and stained for βIII-tubulin. βIII-tubulin-positive cytoskeletal area was quantified and normalized to total cell area as described above. The same imaging and quantification parameters were applied across all groups.

### Quantification and statistical analysis

Quantitative data are presented as mean ± standard error of the mean (SEM) unless otherwise indicated. The sample size (n), definition of biological replicates, and statistical test used for each experiment are specified in the corresponding figure legends. For animal experiments, n represents biologically independent embryos or mice. For cell-based imaging assays, measurements from individual cells or microscopic fields were averaged within each independently cultured and treated experiment, and the resulting biological-replicate means were used as the statistical units. Comparisons between two independent groups were performed using unpaired two-tailed Student’s t-tests. For comparisons involving more than two groups, homogeneity of variance was assessed using the Brown–Forsythe test. Ordinary one-way ANOVA followed by Tukey’s multiple-comparisons test was used when the equal-variance assumption was satisfied. RIP–qPCR data involving multiple immunoprecipitation conditions and candidate transcripts were analyzed by two-way ANOVA followed by Tukey’s multiple comparisons. Statistical analyses of experimental data were performed using GraphPad Prism (v10.1.2). Differences were considered statistically significant at *p* < 0.05. Statistical significance is denoted as follows: ns, not significant; ∗*p* < 0.05; ∗∗*p* < 0.01; ∗∗∗*p* < 0.001; and ∗∗∗∗*p* < 0.0001.

## Supplemental information

Document S1. Figures S1–S12

Table S1. List of oligos

Table S2. Differential alternative splicing events in SH-SY5Y cells upon RBM42 knockdown

Table S3. Genome-wide mapping of RBM42-RNA interactions by RIP-seq in SH-SY5Y cells

Table S4. Gene-level overlap between reproducible RBM42 RIP-seq targets and differentially spliced genes

Table S5. Differential alternative splicing in mouse brain upon Rbm42 conditional knockout

Table S6. Conserved AS-regulated genes in mouse brain and SH-SY5Y cells

Table S7. Proteomic analysis of RBM42 immunoprecipitates by IP-MS

Table S8. Genome-wide mapping of RBM42-RNA interactions by RIP-seq in E18.5 mouse cortical tissues
