# Supplementary material for: RBM42 couples alternative splicing to cytoskeletal organization to direct cortical development
Source: iScience. 2026 Nov 2;29(11):118059. doi: 10.1016/j.isci.2026.118059 (PMC13756106; doi:10.1016/j.isci.2026.118059)
Supplement: Supplementary file 1 [file mmc1.pdf]

iScience, Volume *29*

Supplemental information

RBM42 couples alternative splicing to cytoskeletal organization to direct cortical development

Bingxin Yang, Qingxin Xu, Weiliang Lu, Xiang Qiu, Yanlu Chen, Runqian Shi, Yiyao Chen, Yuwu Jiang, Yanlin Wang, Jian Wang, Hong Xu, He-Feng Huang, and Jinglan Zhang

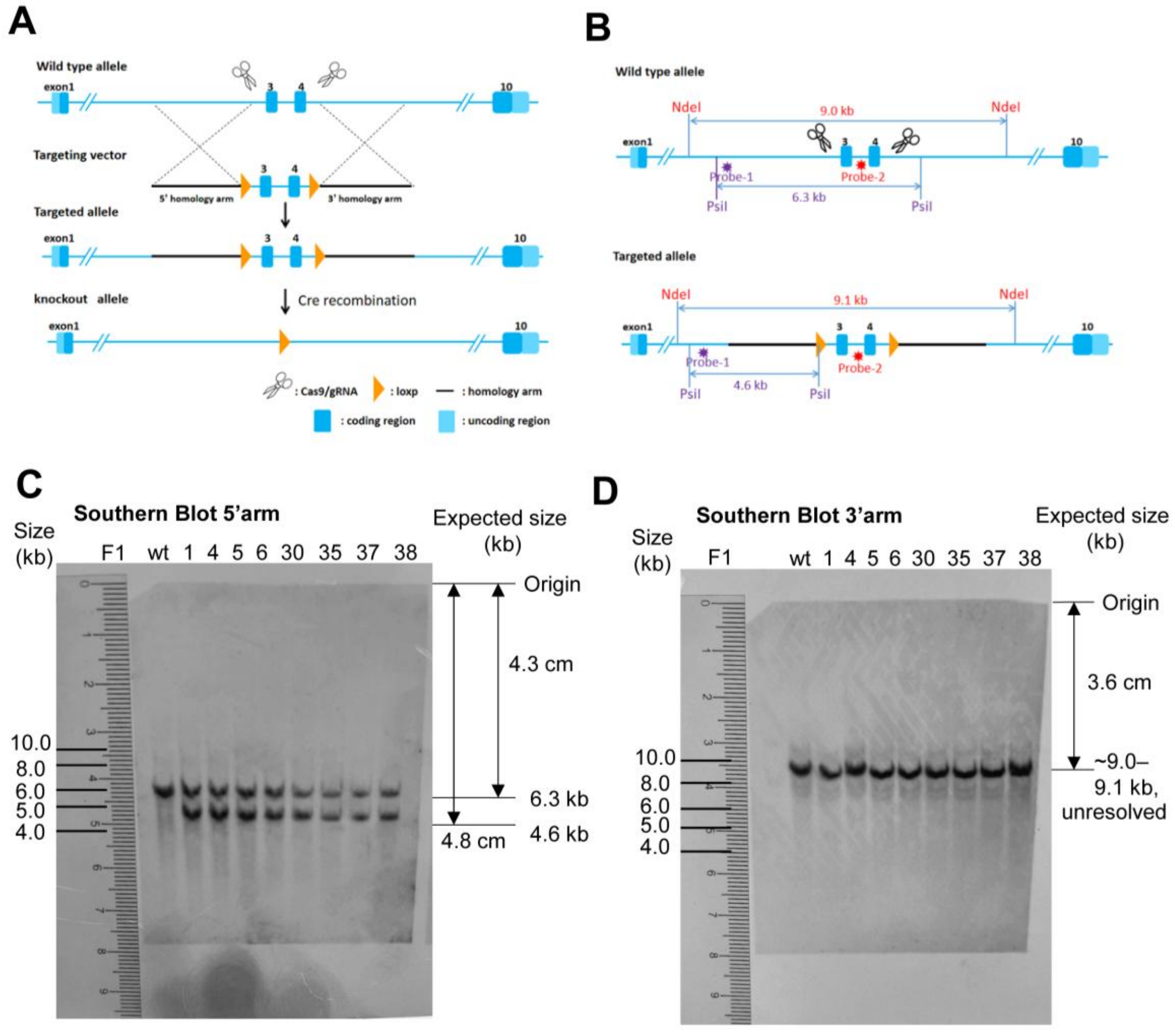

Figure S1 Generation and validation of the *Rbm42* conditional knockout mice.

(A) Strategy used to generate the *Rbm42* floxed allele by CRISPR-Cas9-mediated insertion of loxP sites flanking exons 3–4. After the floxed allele was generated and validated, *Rbm42*$^{flox/flox}$ mice were crossed with *Rbm42*$^{flox/+}$; Nestin-Cre^{+} mice to generate experimental littermates. Control littermates were *Rbm42*$^{flox/flox}$; Nestin-Cre^{-}, whereas cKO littermates were *Rbm42*$^{flox/flox}$; Nestin-Cre^{+}. (B) Southern blot validation strategy and expected fragment sizes. With the 5′ probe, the expected fragments were 4.6 kb for the targeted allele and 6.3 kb for the wild-type (WT) allele. With the 3′ probe, the expected fragments were 9.0 kb for the WT allele and 9.1 kb for the targeted allele. (C) Representative Southern blot results with the 5′ probe, showing both the targeted allele (4.6 kb) and the WT allele (6.3 kb) in heterozygous mice, and only the WT allele (6.3 kb) in the WT control. Correctly targeted recombinants were identified in mice numbered 1, 4, 5, 6, 30, 35, 37, and 38. (D) Southern blot analysis using the 3′ probe. The expected fragment sizes are 9.0 kb for the WT allele and 9.1 kb for the targeted allele. These fragments differ by only 0.1 kb and were not resolved under the conditions used; the signal is therefore indicated as an unresolved ~9.0–9.1 kb band. For panels C and D, DNA size references shown on the left were derived from the DNA markers in the corresponding pre-transfer agarose gels. Marker positions were mapped onto each Southern blot image using the ruler scales, independently for the 5′-arm (C) and 3′-arm (D) analyses. Expected fragment sizes are indicated on the right.

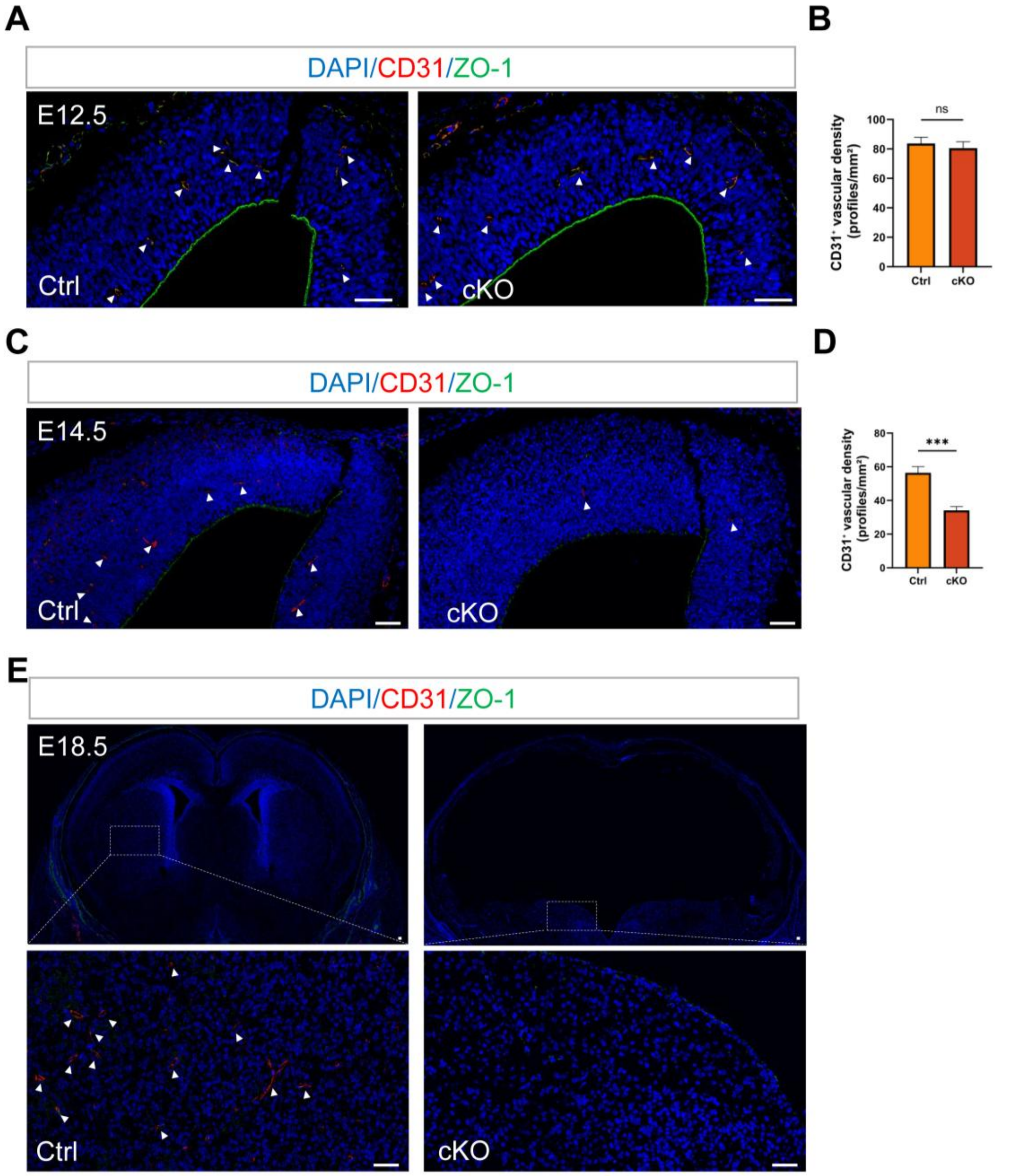

Figure S2 CD31 and ZO-1 staining in *Rbm42* cKO embryonic brains.

(A) Representative immunofluorescence images of CD31, ZO-1, and DAPI in dorsal telencephalic regions from E12.5 control and *Rbm42* cKO embryos (Ctrl, n = 3; cKO, n = 4). Arrowheads indicate representative CD31$^+$ vascular profiles. (B) Quantification of CD31$^+$ vascular profile density in E12.5 dorsal telencephalic regions. CD31$^+$ vascular profiles were manually counted within anatomically matched ROIs and normalized to ROI area in mm^2, yielding the number of CD31$^+$ vascular profiles per mm^2. (C) Representative immunofluorescence images of CD31, ZO-1, and DAPI in cortical regions from E14.5 control and *Rbm42* cKO embryos (Ctrl, n = 5; cKO, n = 7). Arrowheads indicate representative CD31$^+$ vascular profiles. (D) Quantification of CD31$^+$ vascular profile density in E14.5 cortical regions, calculated as described in (B) and expressed as profiles/mm^2. (E) Representative low-magnification and higher-magnification images of CD31, ZO-1, and DAPI staining in E18.5 control and *Rbm42* cKO embryonic brains (n = 5 per genotype). Boxed regions indicate the areas shown at higher magnification. At E18.5, *Rbm42* cKO brains showed extensive cortical parenchymal collapse, precluding reliable whole-cortex vascular density quantification. Data are represented as mean ± SEM; ns, not significant; ***P < 0.001; two-tailed unpaired Student's t-test. Scale bars, 50 μm.

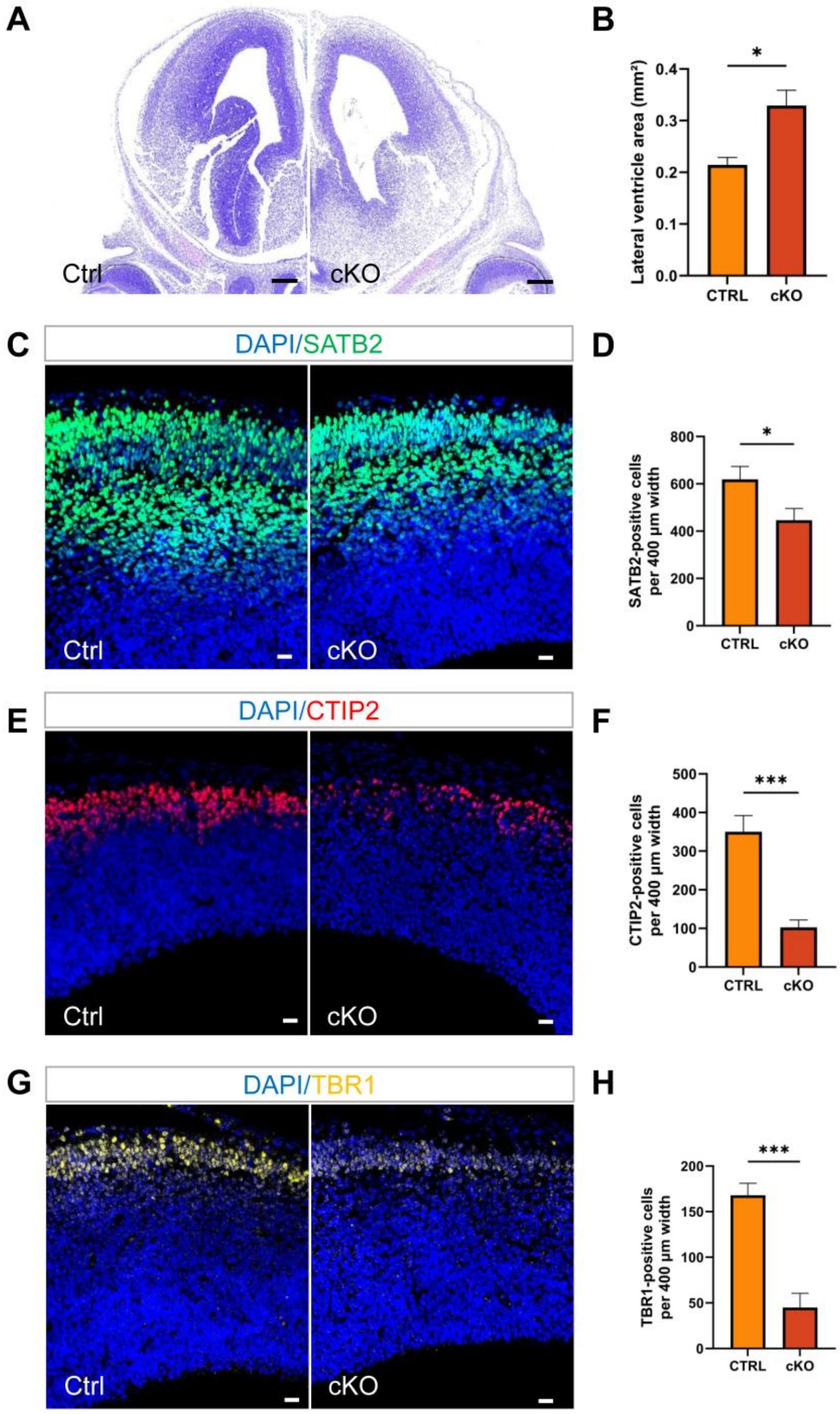

Figure S3 Cortical lamination in E14.5 embryonic brains.

(A) Nissl-stained coronal sections of E14.5 embryonic heads (with skull) showing reduced cortical thickness and enlarged lateral ventricles in *Rbm42* cKO mice compared with Ctrl littermates. Scale bar, 200 μm. (B) Quantification of lateral ventricle area in coronal sections of E14.5 control and *Rbm42* cKO embryos. (C) Immunofluorescence staining of coronal sections for layer-specific markers SATB2 (upper layers) at E14.5. Scale bar, 20 μm. (D) Quantification of cells expressing SATB2 per 400 μm in each section. (E) Immunofluorescence staining of coronal sections for layer-specific markers CTIP2 (deep layers) at E14.5. Scale bar, 20 μm. (F) Quantification of cells expressing CTIP2 per 400 μm in each section. (G) Immunofluorescence staining of coronal sections for layer-specific markers TBR1 (deepest layer/subplate) at E14.5. Scale bar, 20 μm. (H) Quantification of cells expressing TBR1 per 400 μm in each section. For quantifications in (B), (D), (F), and (H), Ctrl, n = 5 embryos; cKO, n = 7 embryos. Data are represented as mean ± SEM. **P* < 0.05, ****P* < 0.001; two-tailed unpaired Student's t-test.

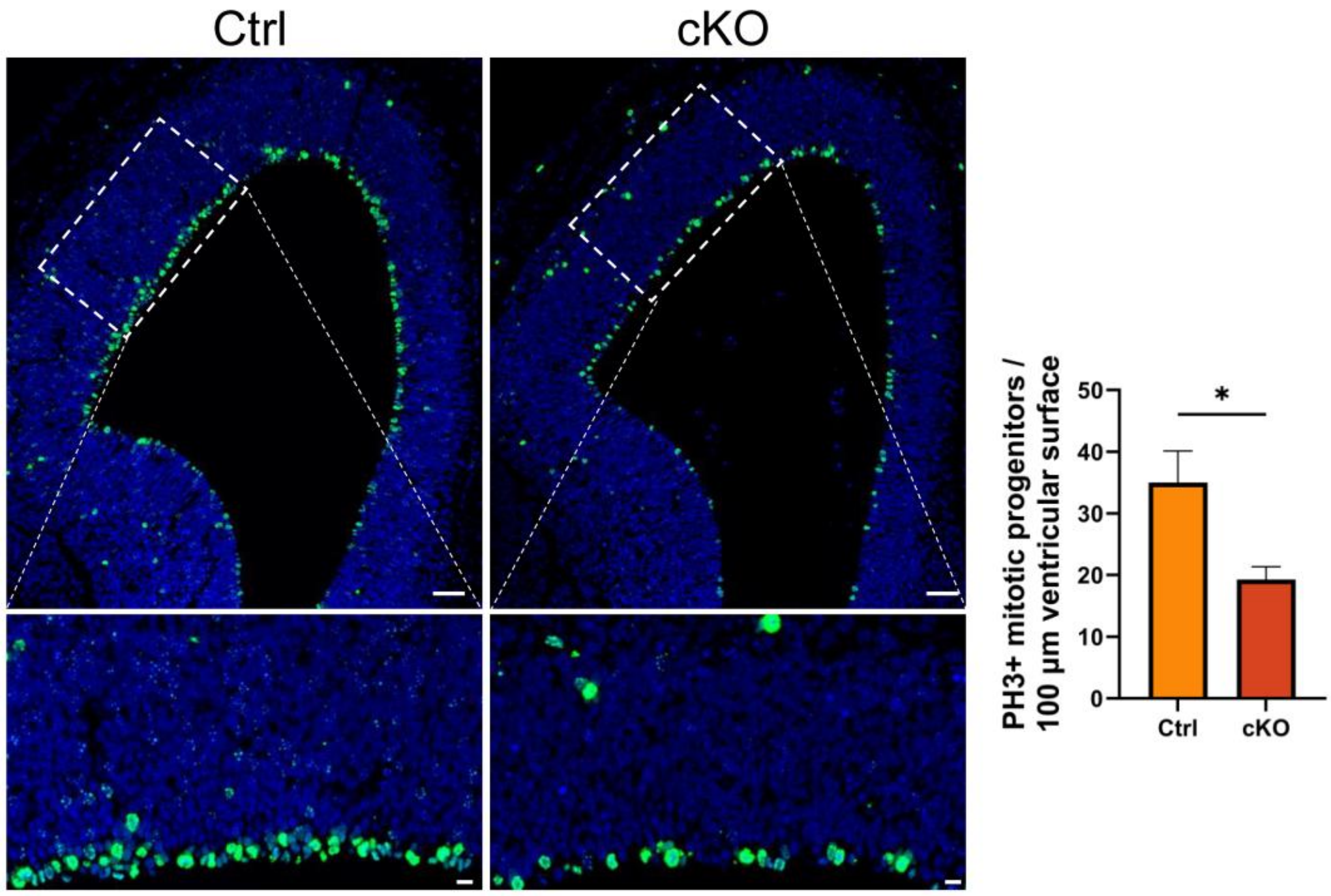

Figure S4 Reduced mitotic progenitor activity in E12.5 *Rbm42* cKO cortices.

Representative images of phospho-histone H3 (PH3) immunostaining in coronal sections from E12.5 control and *Rbm42* cKO embryonic cortices. High-magnification views show PH3-positive mitotic progenitors lining the ventricular surface. Quantification of PH3-positive mitotic cells normalized to ventricular surface length revealed a significant reduction in apical mitotic progenitors in *Rbm42* cKO cortices. Three control embryos and four cKO embryos were analyzed using one anatomically matched section per embryo; embryo-level values were used for inference. Data are presented as mean ± SEM. **P* < 0.05; two-tailed unpaired Student's t-test. Scale bars, low magnification, 200 μm; high magnification, 20 μm.

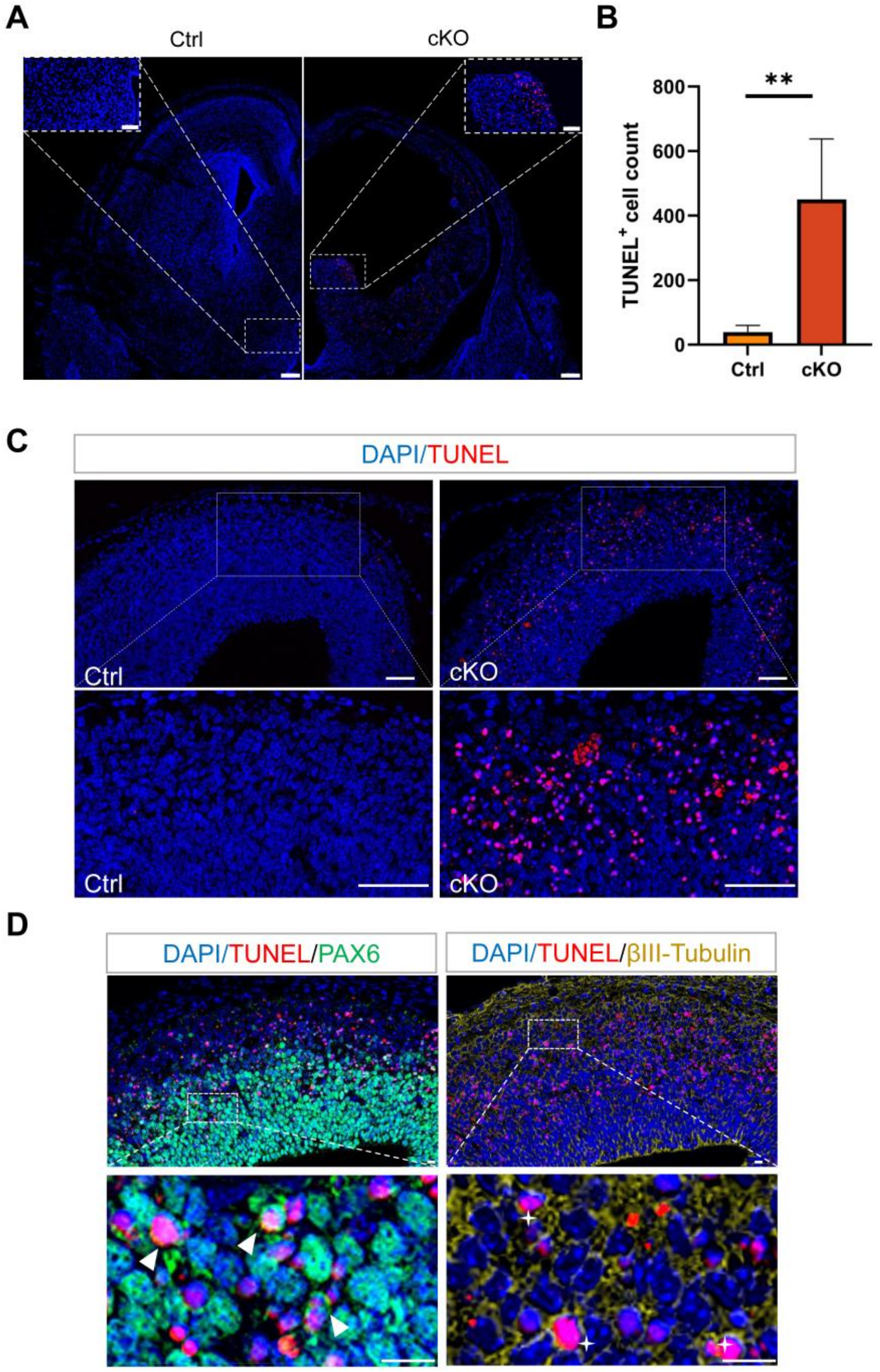

Figure S5 Cortical apoptosis in *Rbm42* cKO embryonic brains.

(A) Representative TUNEL staining of coronal sections from E18.5 control and *Rbm42* cKO brains. White dashed boxes indicate regions shown at higher magnification. Scale bars, 200 μm (overview) and 100 μm (inset). (B) Quantification of TUNEL-positive cells in the cortical ventricular and subventricular zones of E18.5 embryos. Data are represented as mean ± SEM (n = 5 embryos per genotype). (C) Representative TUNEL staining in E14.5 control and *Rbm42* cKO cortices. DAPI labels nuclei. Boxed regions are shown at higher magnification below. Scale bars, 50 μm. (D) Representative TUNEL/PAX6/βIII-tubulin immunofluorescence staining in E14.5 *Rbm42* cKO cortex. Arrowheads indicate TUNEL-positive nuclei co-labeled with PAX6. Asterisks indicate representative TUNEL-positive cells located within βIII-tubulin-rich cortical regions. Scale bars, 10 μm. $^{**}P < 0.01$; two-tailed unpaired Student's t-test.

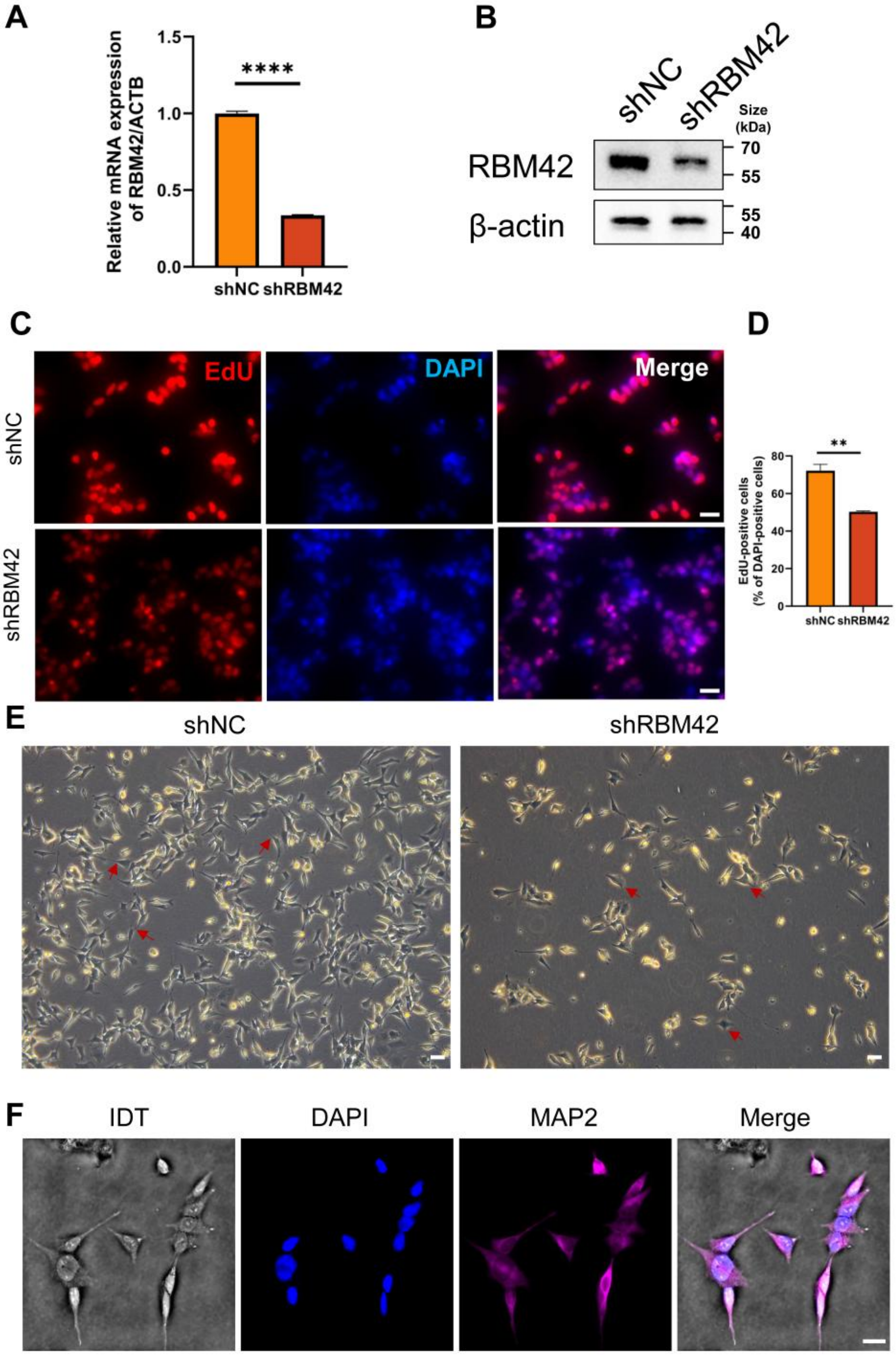

Figure S6 RBM42 depletion impairs proliferation and neurite growth of SH-SY5Y cells.

(A) Quantitative RT–PCR showing reduced *RBM42* mRNA in shRBM42 cells compared with shNC. *RBM42* mRNA levels were normalized to *ACTB*. (B) Western blot analysis of RBM42 protein levels in shNC and shRBM42 cells. β-actin served as a loading control. (C) Representative images of EdU incorporation in SH-SY5Y cells stably transduced with lentiviral vectors expressing shNC or shRBM42 after a 2-h EdU pulse. Nuclei were counterstained with DAPI. Scale bar, 50 μm. (D) Quantification of EdU-positive cells, expressed as a percentage of total DAPI-stained nuclei. (E) Phase-contrast images of SH-SY5Y cells stably expressing control shRNA (shNC) or RBM42-targeting shRNA (shRBM42). Red arrows indicate representative cells; arrowed shNC cells show relatively long neurite-like projections, whereas arrowed shRBM42 cells show shorter projections. Scale bar, 25 μm. (F) Colocalization of neurite-like structures visualized by intensity diffraction tomography with MAP2 immunofluorescence. Scale bar, 20 μm. Data in (A) and (D) are represented as mean ± SEM from three independent biological experiments (n = 3). Statistical significance in (A) and (D) was determined by a two-tailed unpaired Student's t-test. $^{**}P < 0.01$; $^{****}P < 0.0001$.

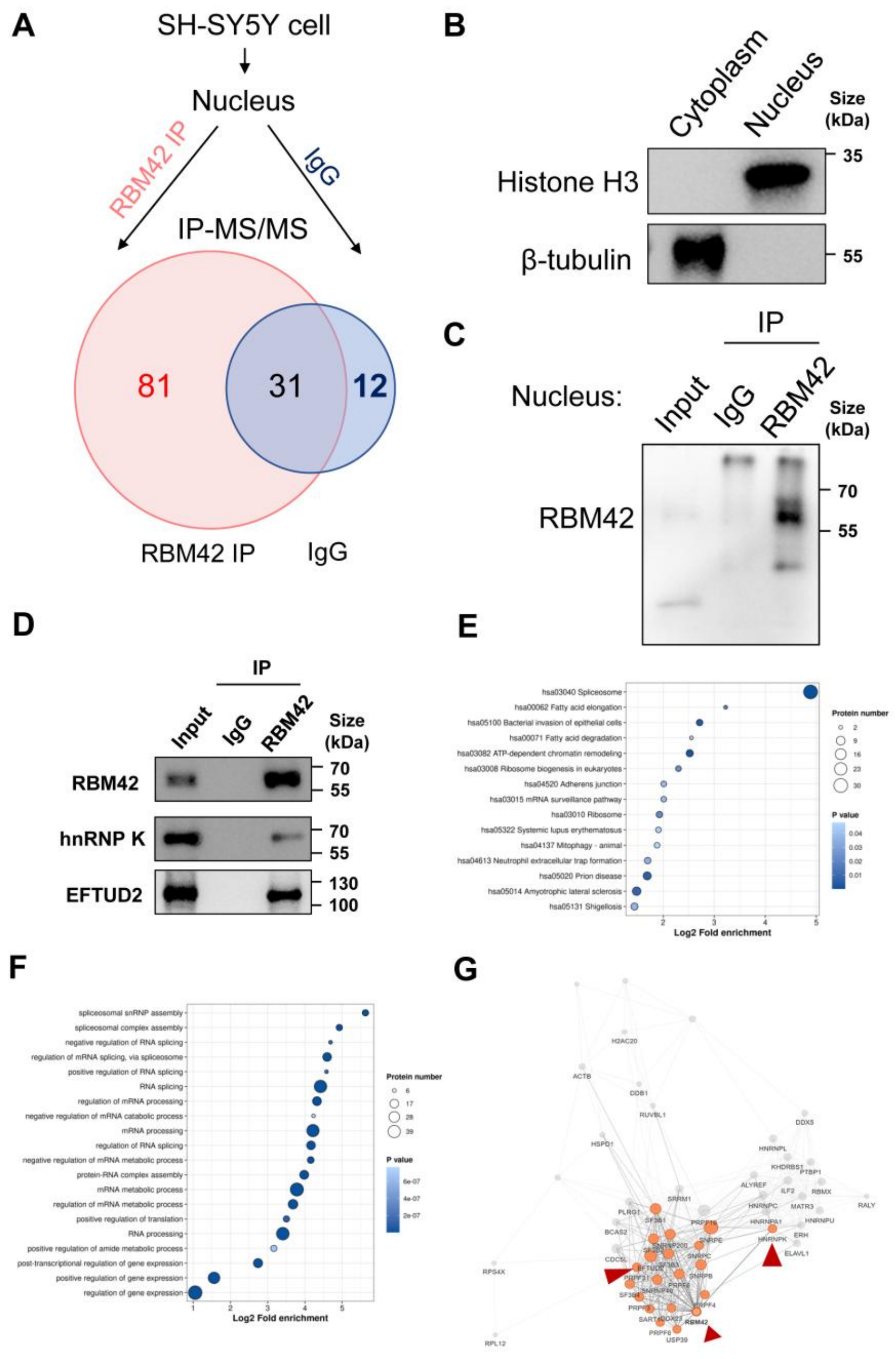

Figure S7 Exploratory proteomic analysis of RBM42 immunoprecipitates.

(A) Schematic of the immunoprecipitation–mass spectrometry (IP–MS/MS) workflow. Nuclear extracts from SH-SY5Y cells were immunoprecipitated with anti-RBM42 antibody or control IgG, followed by liquid chromatography–tandem mass spectrometry (LC–MS/MS). Venn diagram shows 112 proteins detected with RBM42 antibody, 43 with IgG, and 31 common proteins (see Table S7A–S7C). (B) Western blot analysis validating nuclear fractionation. Histone H3 and β-tubulin were detected in the nuclear and cytoplasmic fractions, respectively. The 35-kDa label indicates a molecular-weight marker position above the Histone H3 band. (C) IP-WB confirming efficient immunoprecipitation of RBM42. Input lysate, IgG control IP, and RBM42 IP samples were grouped. (D) Co-immunoprecipitation (Co-IP) validation of RBM42 interactions. SH-SY5Y lysates were immunoprecipitated with anti-RBM42 antibody or control IgG and analyzed by western blotting for hnRNP K, EFTUD2 and RBM42. Input, 8% of total lysate. (E) Kyoto Encyclopedia of Genes and Genomes (KEGG) pathway enrichment analysis of candidate RBM42-associated proteins identified by exploratory IP–MS/MS. (F) Gene Ontology (GO) biological process enrichment analysis of these candidate proteins. In (E) and (F), dot color indicates the enrichment *p* value, and dot size indicates the number of candidate proteins associated with each term. (G) Protein–protein interaction (PPI) network of the candidate RBM42-associated proteins; RBM42, hnRNP K, and EFTUD2 are indicated by red arrows.

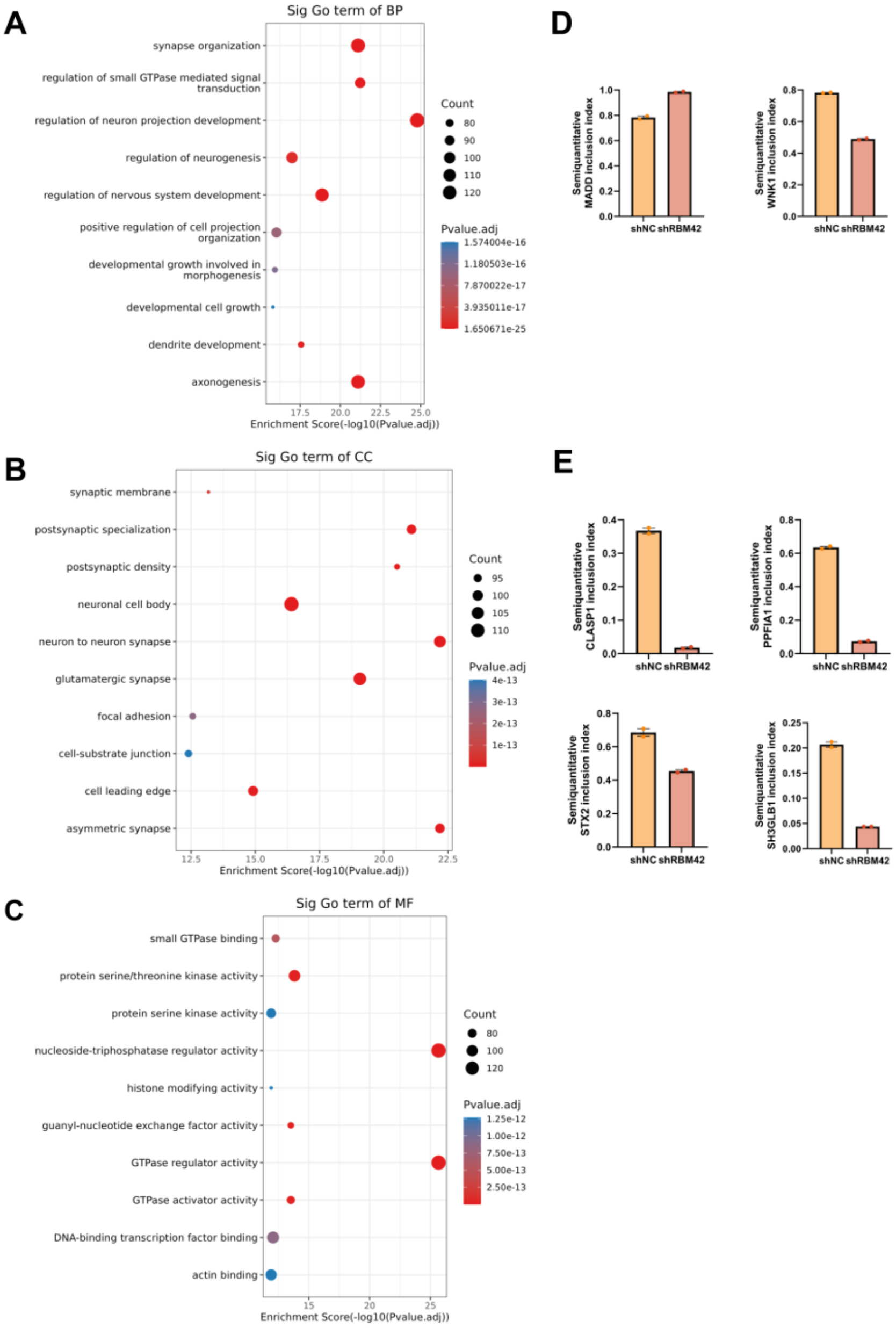

Figure S8 GO enrichment analysis and RT–PCR densitometric quantification of RBM42-bound RNAs identified by RIP–seq in SH-SY5Y cells.

(A–C) GO enrichment of genes associated with reproducible RBM42-binding peaks across three independent RIP–seq experiments. (A) Enriched terms in the Biological Process category. (B) Enriched terms in the Cellular Component category. (C) Enriched terms in the Molecular Function category. Only terms with an FDR < 0.05 are shown; terms are ranked by gene ratio. In (A–C), dot color indicates the adjusted *p* value, dot size indicates the number of genes associated with each GO term, and the horizontal axis shows $-\log_{10}$(adjusted *p* value). (D–E) Densitometric quantification of the RT–PCR bands shown in Figure 4B and Figure 4C. Band intensities corresponding to exon inclusion and skipping isoforms were measured using ImageJ. A semiquantitative exon-inclusion index was calculated as inclusion-band intensity/(inclusion-band intensity + skipping-band intensity). For *SH3GLB1*, reference-transcript mapping predicts inclusion of the 24-nt alternative exon in the 229-bp product and its absence from the 205- and 166-bp products; the index was therefore calculated as I229/(I229 + I205 + I166), where I denotes band intensity. These endpoint-gel ratios were used for relative comparisons and were not interpreted as absolute PSI values. Data in (D) and (E) are presented as mean ± SEM. Data points represent independent biological replicates (n = 2).

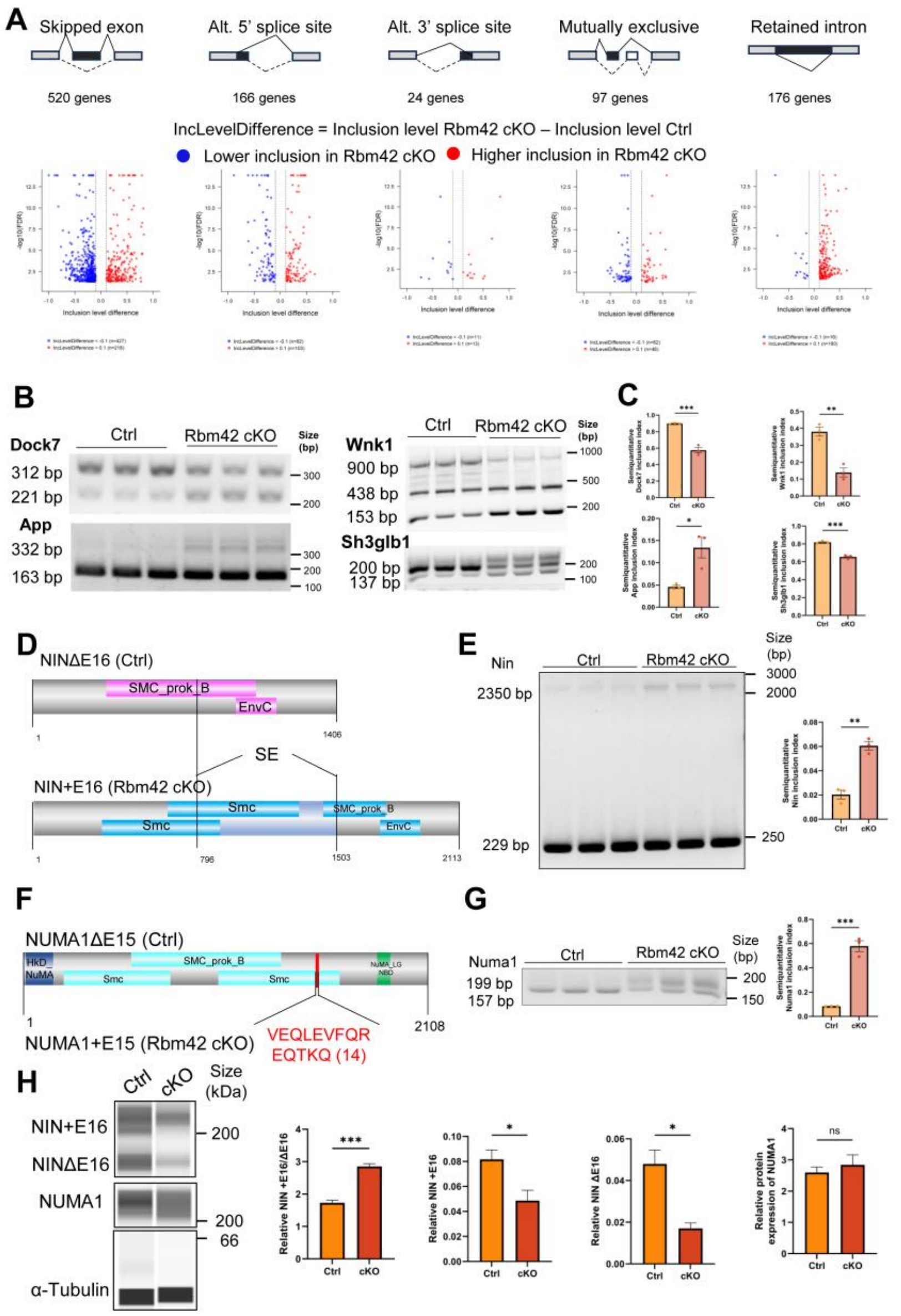

Figure S9 Differential splicing analysis in Rbm42 cKO mouse brains.

(A) AS categories and the number of genes with at least one event in each category (top) and volcano plots (bottom). Differential events were defined by FDR < 0.05 and |IncLevelDifference| > 0.1; positive values indicate higher inclusion in cKO than control samples (Table S5). (B–C) RT–PCR validation of SE events in *Dock7*, *App*, *Wnk1*, and *Sh3glb1* (B) and ImageJ densitometry (C). Inclusion indices were calculated as inclusion/(inclusion + skipping) band intensity. For *Wnk1*, reference-transcript mapping predicts that only the 900-bp product contains the 462-nt exon; the index was calculated as I900/(I900 + I438 + I153), where I denotes band intensity. These indices are semiquantitative, not absolute PSI values. Dots indicate biological replicates; data are mean ± SEM. (D, F) NINΔE16/NIN+E16 (D) and NUMA1ΔE15/NUMA1+E15 (F) protein models. The included sequences comprise 707 and 14 amino acids, respectively, and overlap CDD-predicted SMC-superfamily matches. (E, G) Semiquantitative RT–PCR showing increased inclusion of *Nin* exon 16 (E) and *Numa1* exon 15 (G) in cKO brains. (H) Compass-generated capillary-western virtual blots and quantification of NIN isoforms and total NUMA1 in embryonic cortical lysates. Molecular-weight labels indicate Compass-calibrated reference positions (kDa). Protein peak areas were normalized to α-tubulin; the NIN+E16/NINΔE16 ratio is also shown. Data are mean ± SEM (n = 3 independent embryos per genotype; one lysate per embryo). Unpaired two-tailed Student's t-test: *P < 0.05, **P < 0.01, ***P < 0.001; ns, not significant.

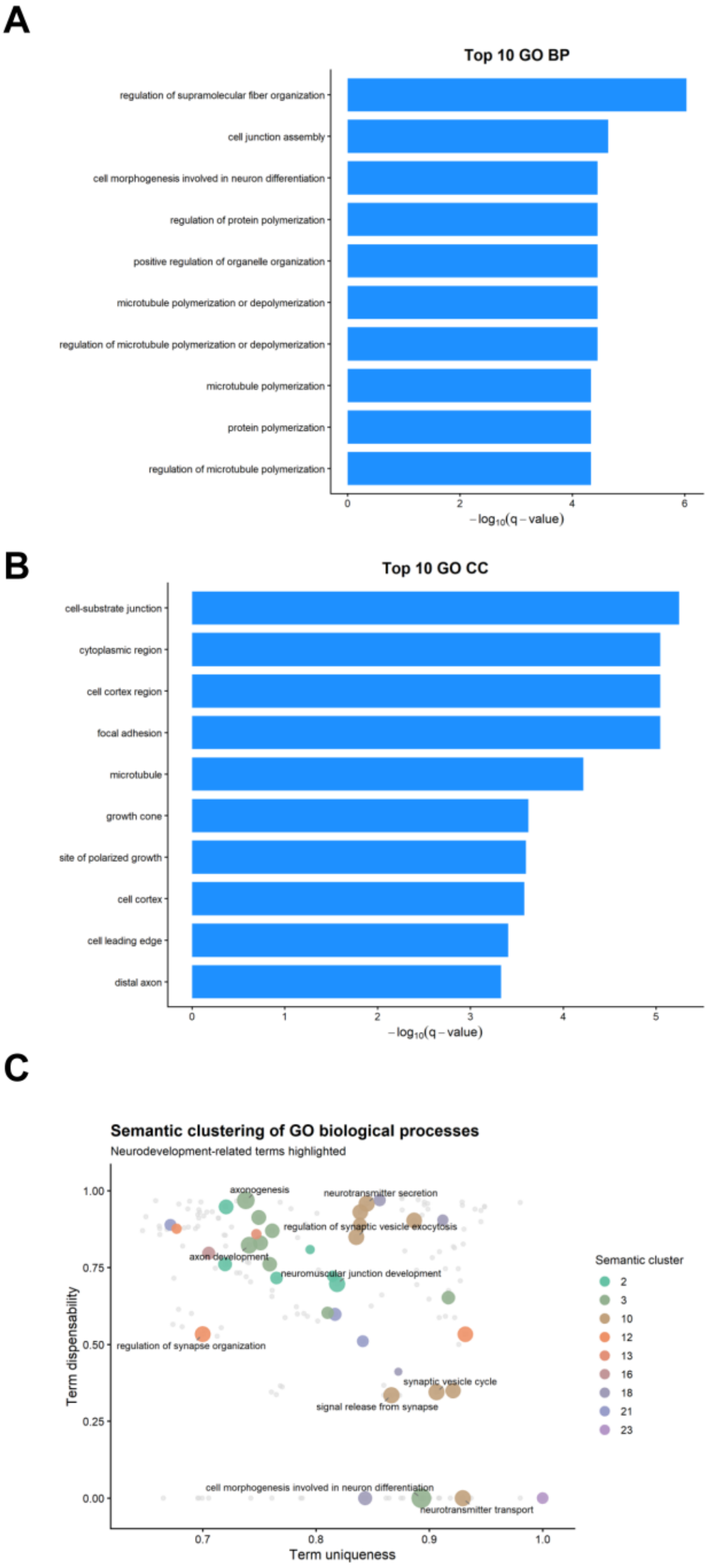

Figure S10 GO enrichment and semantic clustering of conserved AS-regulated genes.

(A–B) Gene Ontology enrichment analysis of the 87 human orthologs representing gene pairs with significant AS changes in both the human and mouse datasets. (A) Biological Process. (B) Cellular Component. Terms with q value < 0.05 and at least five input genes were retained. The ten most significant terms ranked by q value are shown, and the x-axis represents −log10(q value). (C) Semantic similarity–based summarization of enriched GO biological processes. Enriched GO biological process terms were summarized using rrvgo to reduce redundancy based on semantic similarity. Each point represents an enriched GO term plotted by term uniqueness (x-axis) and term dispensability (y-axis). Grey points denote all analyzed enriched terms, whereas colored points indicate neurodevelopment-related terms identified by keyword-based annotation. Colors represent semantic clusters defined by rrvgo. Point size corresponds to enrichment significance (–log10 adjusted *P* value). The ten most significantly enriched neurodevelopment-associated terms are labeled.

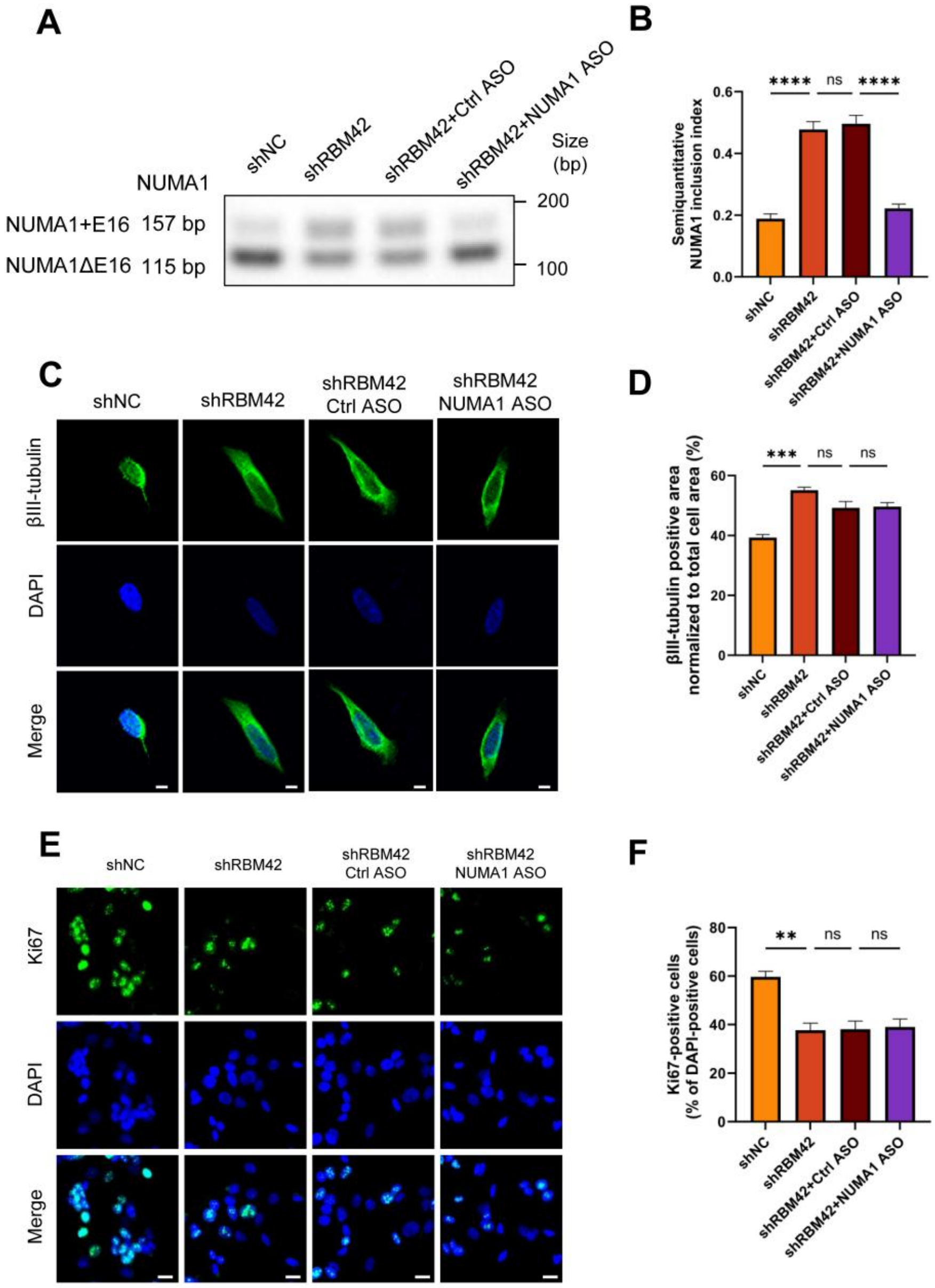

Figure S11 Effects of NUMA1 exon 16 splice correction on βIII-tubulin cytoskeletal organization and cell proliferation.

(A) RT–PCR analysis of *NUMA1* exon 16 splicing in shNC, shRBM42, shRBM42 + Ctrl ASO, and shRBM42 + *NUMA1* ASO SH-SY5Y cells. Upper band, *NUMA1*+E16; lower band, *NUMA1*ΔE16. (B) Semiquantitative *NUMA1* exon 16 inclusion index from the RT–PCR products shown in (A), calculated as *NUMA1*+E16 band intensity divided by the summed *NUMA1*+E16 and *NUMA1*ΔE16 band intensities. (C) Representative immunofluorescence images of βIII-tubulin and DAPI in the indicated cells. Scale bar, 2 μm. (D) Quantification of βIII-tubulin-positive area normalized to total cell area. Single-cell ROIs were manually delineated, and βIII-tubulin-positive area was measured from thresholded βIII-tubulin images (n = 3 independent experiments). For each independent experiment, 5–20 randomly selected fields were analyzed per condition. (E) Representative immunofluorescence images of Ki67 and DAPI in the indicated cells. Scale bar, 20 μm. (F) Quantification of the proportion of Ki67-positive cells among DAPI-positive cells. For each independent experiment, three randomly selected fields were analyzed per condition (n = 3 independent experiments). *NUMA1* ASO treatment did not significantly restore the reduction in Ki67-positive cells induced by RBM42 depletion. Data in (B), (D), and (F) are represented as mean ± SEM. Statistical significance in (B), (D), and (F) was determined by ordinary one-way ANOVA followed by Tukey's multiple-comparisons test. ns, not significant; ** $P < 0.01$; *** $P < 0.001$; **** $P < 0.0001$.

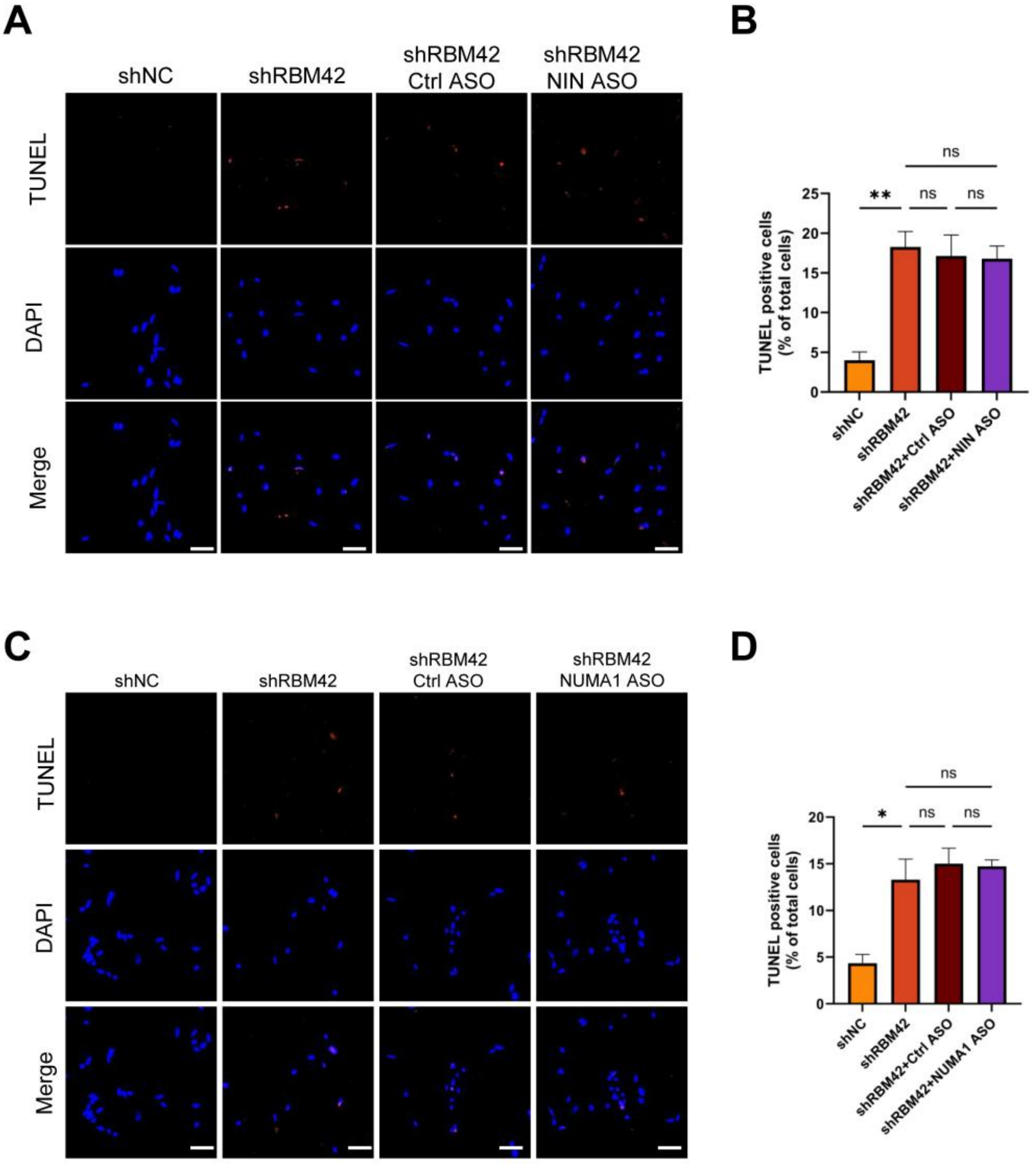

Figure S12 Assessment of apoptosis following *NIN* and *NUMA1* splicing correction in RBM42-deficient SH-SY5Y cells.

(A) Representative images of TUNEL staining in SH-SY5Y cells treated with the indicated conditions. Stable RBM42-knockdown cells (shRBM42) were treated with Ctrl ASO or *NIN* exon 18-targeting ASO, and apoptosis was assessed by TUNEL staining. Nuclei were counterstained with DAPI. Scale bar, 50 μm. (B) Quantification of the TUNEL-positive fraction shown in (A), expressed as the percentage of total DAPI-positive nuclei. Three randomly selected fields per condition were analyzed in each of three independent experiments; all DAPI-positive nuclei within each field were counted, field values were averaged within each experiment, and biological-replicate means were used for inference. (C) Representative images of TUNEL staining in SH-SY5Y cells treated with Ctrl ASO or *NUMA1*-targeting ASO under RBM42-deficient conditions. Nuclei were counterstained with DAPI. Scale bar, 50 μm. (D) Quantification of the TUNEL-positive fraction in (C). The same field-sampling and biological-replicate averaging procedure described for (B) was used. Data are represented as mean ± SEM. Statistical significance was determined by one-way ANOVA followed by Tukey's multiple-comparisons test. $^{*}P < 0.05$, $^{**}P < 0.01$; ns, not significant.
